# Bifunctional crosslinking ligands for transthyretin

**DOI:** 10.1098/rsob.150105

**Published:** 2015-09-23

**Authors:** P. Patrizia Mangione, Stéphanie Deroo, Stephan Ellmerich, Vittorio Bellotti, Simon Kolstoe, Stephen P. Wood, Carol V. Robinson, Martin D. Smith, Glenys A. Tennent, Robert J. Broadbridge, Claire E. Council, Joanne R. Thurston, Victoria A. Steadman, Antonio K. Vong, Christopher J. Swain, Mark B. Pepys, Graham W. Taylor

**Affiliations:** 1 Wolfson Drug Discovery Unit, Centre for Amyloidosis and Acute Phase Proteins, Division of Medicine, University College London, Royal Free Campus, Rowland Hill Street, London NW3 2PF, UK; 2 Department of Molecular Medicine, Institute of Biochemistry, University of Pavia, Pavia, Italy; 3 Chemistry Research Laboratory, University of Oxford, 12 Mansfield Road, Oxford OX1 3TA, UK; 4 Peptide Protein Research Ltd, Claylands Road, Bishops Waltham, Southampton, Hampshire SO32 1QD, UK; 5 Selcia Ltd, Fyfield Road, Ongar, Essex CM5 0GS, UK; 6 Cambridge MedChem Consulting, 8 Mangers Lane, Duxford, Cambridge CB22 4RN, UK

**Keywords:** amyloidosis, transthyretin, crosslinking, plasma clearance

## Abstract

Wild-type and variant forms of transthyretin (TTR), a normal plasma protein, are amyloidogenic and can be deposited in the tissues as amyloid fibrils causing acquired and hereditary systemic TTR amyloidosis, a debilitating and usually fatal disease. Reduction in the abundance of amyloid fibril precursor proteins arrests amyloid deposition and halts disease progression in all forms of amyloidosis including TTR type. Our previous demonstration that circulating serum amyloid P component (SAP) is efficiently depleted by administration of a specific small molecule ligand compound, that non-covalently crosslinks pairs of SAP molecules, suggested that TTR may be also amenable to this approach. We first confirmed that chemically crosslinked human TTR is rapidly cleared from the circulation in mice. In order to crosslink pairs of TTR molecules, promote their accelerated clearance and thus therapeutically deplete plasma TTR, we prepared a range of bivalent specific ligands for the thyroxine binding sites of TTR. Non-covalently bound human TTR–ligand complexes were formed that were stable *in vitro* and *in vivo*, but they were not cleared from the plasma of mice *in vivo* more rapidly than native uncomplexed TTR. Therapeutic depletion of circulating TTR will require additional mechanisms.

## 1. Background

Systemic amyloidosis is a serious disease caused by the extracellular deposition of circulating globular proteins as abnormal, insoluble fibrils in the viscera, blood vessel walls and connective tissues. It is usually fatal, causing about one per thousand deaths in developed countries [1]. Wild-type transthyretin (TTR) is a normal plasma protein that circulates as a tetramer of four identical subunits and acts as a transporter for thyroid hormone and retinol binding protein. It is inherently amyloidogenic and forms microscopic amyloid deposits in almost all individuals aged over 80 years [2]. Massive deposits in the heart can also occur, causing fatal senile cardiac TTR amyloidosis [3]. The inherent amyloidogenicity of wild-type TTR is markedly enhanced by most of the reported more than 100 different point mutations that encode single residue substitutions in the TTR sequence. These mutations cause autosomal dominant adult-onset hereditary amyloidosis, a universally fatal condition affecting about 10 000 patients worldwide. The usual clinical presentation is familial amyloid polyneuropathy, with predominant peripheral and autonomic neuropathy, but there is commonly also serious involvement of the heart, kidneys and eyes. The condition typically presents after the causative gene has been transmitted to the proband's offspring, ensuring persistence of this devastating disease. Amyloidogenic mutations occur in all ethnic groups, but by far the most common, Val30Met, clusters in three geographical foci: Northern Portugal, Northern Sweden and parts of Japan. A common amyloidogenic variant in the UK and Eire is Thr60Ala. TTR amyloidosis predominantly affecting the heart is particularly associated with the Val122Ile variant, which is very rare in Caucasians but is carried by 4% of African-Americans [4]. It is the second most common pathogenic mutation in that population after sickle cell haemoglobin. Cardiac TTR amyloidosis presents as progressive, ultimately fatal, heart failure owing to restrictive cardiomyopathy, is rarely suspected and is usually misdiagnosed as coronary heart disease.

There was no effective treatment for TTR amyloidosis until orthotopic liver transplantation was introduced in 1991 [5]. Circulating TTR is synthesized mainly by hepatocytes, and liver transplantation removes the source of the amyloidogenic variant TTR in the plasma and replaces it with wild-type TTR. However, the procedure is available for only a minority of patients and optimal results are obtained early in the disease. Furthermore, patients with mutations other than Val30Met have developed rapidly progressive cardiac amyloidosis after transplantation [6], presumably because of the natural amyloidogenicity of wild-type TTR. In patients with predominant cardiac amyloid, heart transplantation is a possible option, but most are too old and are not acceptable recipients of scarce donor organs. Nevertheless, the efficacy of liver transplantation in arresting amyloid deposition for patients with the Val30Met mutation demonstrated the potential efficacy of plasma TTR depletion as a treatment for TTR amyloidosis.

One approach to TTR depletion is direct inhibition of hepatic synthesis, and this has lately been successfully demonstrated by ISIS Pharmaceuticals, Inc. [7] and by Alnylam Pharmaceuticals [8] using pharmaceutical antisense oligonucleotide and siRNA approaches, respectively. Alternatively, the concentration of circulating TTR could be lowered by increasing its clearance. Native TTR is mainly catabolized in the liver, but also in muscle, skin, kidney, adipose tissue and the gastrointestinal tract [9]. Fibroblasts have recently been shown to remove TTR aggregates [10]. Disruption and clearance of TTR amyloid deposits is also a potential therapeutic approach [11]; however, prevention of amyloid deposition would be preferable. We therefore sought to modify the native structure of circulating TTR to promote its accelerated clearance from the plasma and catabolism leading to TTR depletion. We have previously shown that clearance of human serum amyloid P component (SAP) is dramatically increased by non-covalently crosslinking pairs of SAP molecules with a palindromic bifunctional small molecule ligand, hexanoyl bis(d-proline) (CPHPC), that is specifically bound by SAP [12]. Plasma SAP remains at extremely low concentration for as long as the drug is administered [13]. We therefore designed a range of bifunctional ligands to be specifically and non-covalently bound by human TTR, crosslinking pairs of the native protein molecules in stable complexes that, similarly to the SAP–CPHPC complexes, would, we hoped, be recognized as abnormal and promptly cleared.

## 2. Material and methods

Isolated human TTR was purchased from Scipac Ltd, Kent, UK and dissolved in phosphate-buffered saline (PBS). Sodium [^125^I] iodide and ^125^I-thyroxine were purchased from Perkin Elmer, Seer Green, UK. TTR was radiolabelled with ^125^I using *N*-bromosuccinimide and sodium [^125^I] iodide in PBS for 10–15 s and purified on a PD10 desalting column (Bio-Rad, Hemel Hempstead, UK) [14]; ^125^I-TTR was mixed with native TTR as a marker for *in vivo* clearance experiments. Chemically crosslinked TTR was generated by treatment of TTR (10 mg ml^−1^) with a 50-fold molar excess of *N*-(3-dimethylaminopropyl)-*N*'-ethylcarbodiimide hydrochloride (EDC, Sigma-Aldrich, Gillingham, Dorset, UK) in water at pH 5.

**Figure 1.**
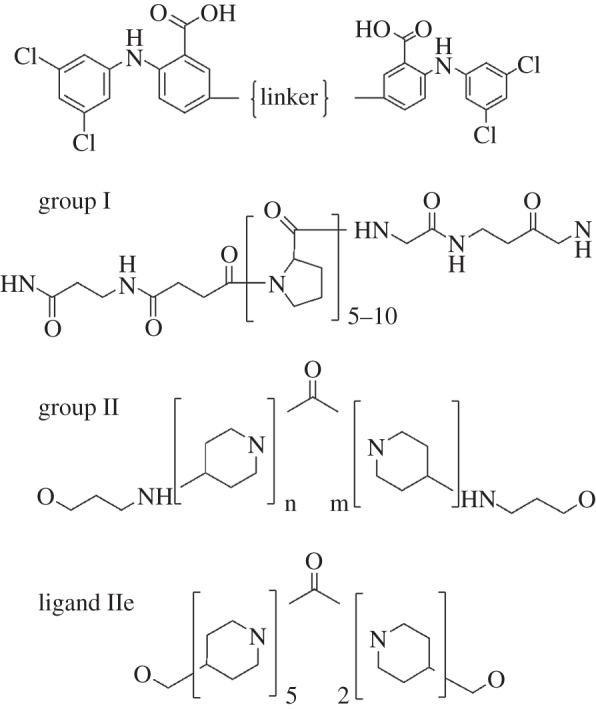
Bifunctional ligands for crosslinking TTR tetramers based on the dichlorophenylaminobenzoic acid head group with either polyproline (group I) or polypiperidine (group II) linkers. Ligand IIe has a modified ether linkage in place of the aminopropoxy linkage.

Two sets of bivalent potential TTR crosslinking ligands were prepared by coupling two molecules of the head group 2-(3,5-dichlorophenylamino)-5-methoxy-benzoic acid with either polyproline (group I) or polypiperidine (group II) linkers. The structures of the ligands are shown in figure 1. The head group and group II ligands were synthesized by Selcia Ltd, Ongar, Essex, UK; full details are in the electronic supplementary material. The group I peptide succinimido-(Pro)*_n_*-Gly-OH (*n* = 5–10) ligands were prepared by Peptide Protein Research Ltd, Southampton, UK using Fmoc peptide chemistry on a Symphony automated peptide synthesizer (Protein Technologies, Manchester, UK). The peptides were cleaved from the solid support and purified by RP-HPLC. The methyl ester protected and amino functionalized 5-amino-2-(3,5-dichlorophenylamino)benzoic acid head group (supplied by Selcia Ltd) was reacted to each peptide using a 2.5-fold molar excess of head group, and monitored by mass spectrometry for addition of head group to both ends of the peptide. The crude product was lyophilized and the methyl ester saponified by the addition of lithium hydroxide in methanol/water. The polyproline ligands were purified by RP-HPLC, and analysed by LC–MS. Further details of synthesis and purification methods are available in the electronic supplementary material. Ligands were dissolved in DMSO at a concentration of 5–10 mM and stored at −30°C until used. For binding experiments, TTR was used at a concentration of 200 µg ml^−1^ (3.6 µM tetramer) in PBS, which is within the normal range of circulating TTR. All ligands were examined using a TTR/^125^I-thyroxine displacement assay as previously described [15,16]. TTR/ligand complexes were prepared by incubation of TTR (1 mg ml^−1^ in PBS) with a 0- to fivefold molar excess of ligand for up to 18 h at room temperature. The concentration of DMSO was kept below 2%. Complexes were gel filtered at 0.5 ml min^−1^ on a Superdex 200 column in either PBS or 150 mM ammonium bicarbonate (pH 7.6) using an Akta Explorer (GE Healthcare, Amersham, Bucks, UK). Molecular weight markers used to calibrate the column were: ribonuclease A (13 700) 18.7 ml, carbonic anhydrase (29 000) 15.8 ml, conalbumin (75 000) 13.7 ml, ferritin (440 000) 9.8 ml and blue dextran (2 000 000) 8.2 ml. Binding of ^125^I-thyroxine by TTR oligomers was examined by native gel electrophoresis in 1% agarose gel in barbitone–calcium buffer (180 V, 1 h) [17] followed by autoradiographic analysis (Typhoon scanner, GE Healthcare). Analytical ultracentrifugation was carried out in the An-50 Ti rotor (XL-I ultracentrifuge, Beckman-Coulter).

TTR/ligand complexes were analysed by nanoflow electrospray mass spectrometry. Mass spectra were recorded (LCT mass spectrometer with Z-spray source; Waters, Elstree, Herts, UK) with capillary voltage of 1.7 kV, sample cone at 80 V, extraction cone at 5 V, ion transfer stage pressure at 5.50 millibar, and time of flight analyser pressure at 1.75 × 10^−6^ millibar. For ligand dissociation experiments, the sample cone voltage was increased stepwise to 200 V. Tandem MS was carried out on a QSTAR XL platform (MDS Sciex) with capillary voltage of 1.4 kV, declustering potential at 100 V, focusing potential at 150 V and collision energy up to 120 V. The relevant *m*/*z* range was selected in the second quadrupole and subjected to acceleration in the collision cell. Immediately before analysis, fully reduced recombinant ^15^N-labelled TTR [18] preparations were buffer-exchanged into 20 mM ammonium acetate, pH 7.0 (Micro Bio-Spin 6 column; Bio-Rad). TTR (4.4 µM) in the presence of different molar ratios of ligands or DMSO alone (2.5% v/v final DMSO concentration) were used to monitor ligand binding and formation of TTR oligomers. All spectra were calibrated externally using CsI and processed with MassLynx v. 4.0 (Waters).

*In vivo* clearance of EDC-TTR was measured in wild-type C57BL/6 mice. Clearance of the non-covalent TTR–ligand complexes was determined in TTR-knockout C57BL/6.SPF congenic mice [19] to avoid any possible ligand exchange between mouse and human TTR. Preformed complexes in PBS were injected intravenously into the tail vein and blood samples collected into heparin at 5, 30, 60 and 180 min. Plasma TTR was quantified by electroimmunoassay in 1% w/v agarose gels prepared in barbitone–EDTA buffer pH 8.6 [20] using monospecific rabbit anti-human TTR antiserum (Dako, Ely, Cambridge, UK), and calibrated with isolated pure wild-type TTR (Scipac Ltd). Total ^125^I in whole blood was measured in a Perkin Elmer 2470 Auto gamma counter with results expressed as cpm mg^−1^. Localization of tracer to kidney, spleen and liver was also determined after washing the organs in PBS. Clearance half-lives were estimated with GraphPad Prism 5.03 using a simple exponential decay model.

## 3. Results

### 3.1. Enhanced clearance of covalently crosslinked transthyretin

**Figure 2.**
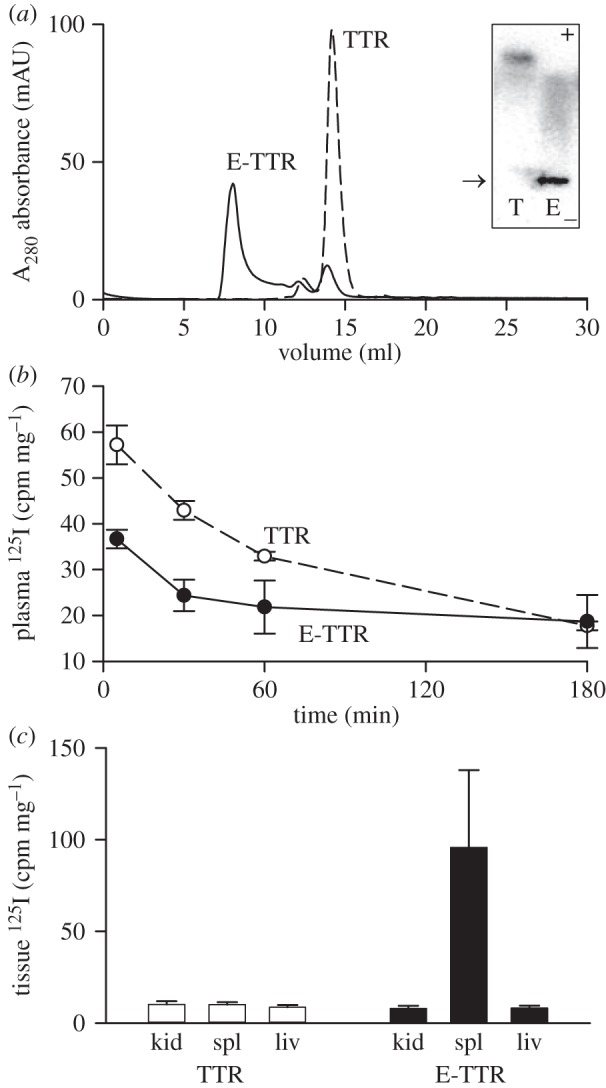
(*a*) A_280_ UV absorbance gel filtration profiles of native TTR (dotted line) and EDC crosslinked TTR (E-TTR, solid line). Native untreated TTR chromatographs as a tetramer on gel filtration eluting at 14.2 ml while EDC crosslinked TTR elutes mainly as high molecular weight oligomers (Ve 7.5 9.0 ml). Both native TTR (T) and EDC-treated TTR (E-TTR) can bind ^125^I-thyroxine as shown in the native gel autoradiogram (inset). Native TTR migrates towards the anode while EDC-TTR remains mostly at the site of sample deposition (arrowed). As some of the aggregates may have reduced thyroxine-binding capacity, the relative intensities in the autoradiogram may not reflect the absolute amounts of material present on the gel. The gel filtration A_280_ UV absorbance profile gives a more accurate representation of relative amounts. (*b*) Clearance of EDC-treated ^125^I-TTR (closed circles) in wild-type mice is faster than for TTR control (open circles); data are expressed as mean and s.d. (*n* = 3). (*c*) ^125^I-TTR is localized in the spleen of animals treated with oligomerized EDC-TTR. Kid, kidney; spl, spleen; liv, liver.

Covalently crosslinked oligomers of TTR were generated using EDC and most of the product eluted in and just after the void volume of the Superdex 200 column (figure 2*a*). The crosslinked TTR retained the capacity to bind ^125^I-thyroxine and after native agarose gel electrophoresis most of the product remained at the origin, well separated from native TTR, with some lower molecular weight oligomers appearing as an anodal smear (figure 2*a*, inset). The electrophoretic and gel filtration data are both consistent with the generation of high-mass oligomers of TTR by EDC treatment. EDC-^125^I-TTR was cleared from the circulation in wild-type mice much more rapidly than ^125^I-TTR. In previous studies, we have found the plasma half-life of native ^125^I-human TTR in wild-type mice to be mean (s.d.), 48.0 (17.6) min, *n* = 7; here, it was 43.5 min with clearance of only 5.5% in the first 5 min. In contrast, 39.4% of the EDC-^125^I-TTR was cleared by 5 min, and clearance of the remainder could not be curve fitted (figure 2*b*). When organs were counted after clearance was complete, there was over 10-fold more radioactivity in the spleens of mice receiving EDC-TTR than those given native control TTR (figure 2*c*).

### 3.2. Preparation and effects of potential crosslinking compounds

A range of potential TTR crosslinking agents were designed from a selection of readily available building blocks that allowed exploration of linker length and flexibility. They were prepared by coupling the TTR-binding head group, 5-amino-2-(3,5-dichlorophenylamino)benzoic acid, to a variety of rigid and non-rigid linkers based on polyproline (Pro) and polypiperidine (Pip) functionalities (figure 1 and table 1). The dichlorophenylaminobenzoic head group was chosen as it had been previously reported by Purkey *et al.* [21] to be avidly bound by TTR in plasma, and was the basis for the development of our TTR superstabilizer, mds84 [16]. Group I ligands contained linear chains of 5–10 proline residues coupled at each end through C6 amides. Group II ligands were polypiperidines linked through a central carbonyl group and further coupled through either 3-aminopropanol or 3-hydroxypropamide. Each ligand was efficiently bound by TTR at the thyroxine binding site with IC_50_ values less than 5 µM in the standard thyroxine displacement assay [16].

**Table 1.** Oligomerization of TTR by bifunctional ligands.

ligand	linker	gel filtration
Ia	Pro5	no crosslinking
Ib Ic Id Ie If	Pro6 Pro7 Pro8 Pro9 Pro10	TTR eluted as a broad peak, consistent with crosslinking in the presence of excess ligand, followed by dissociation on the column. Complexes were not stable to a second gel filtration step
IIa IIb	Pip_3_.CO.Pip_3_ Pip_4_.CO.Pip_1_	more stable crosslinking of pairs of native TTR tetramers than with group I ligands, however complexes still dissociated to individual TTR tetramers on the column
IIc IId	Pip_4_.CO.Pip_4_ Pip_5_.CO.Pip_5_	crosslinking of pairs of TTR molecules to form complexes stable on a repeat gel filtration
IIe	Pip_5_.CO.Pip_2_	TTR eluted as a broad peak, consistent with the formation of larger oligomers which dissociate on the column (ligand IIe contained a modified short ether linkage between the head group and the polypiperidine)

**Figure 3.**
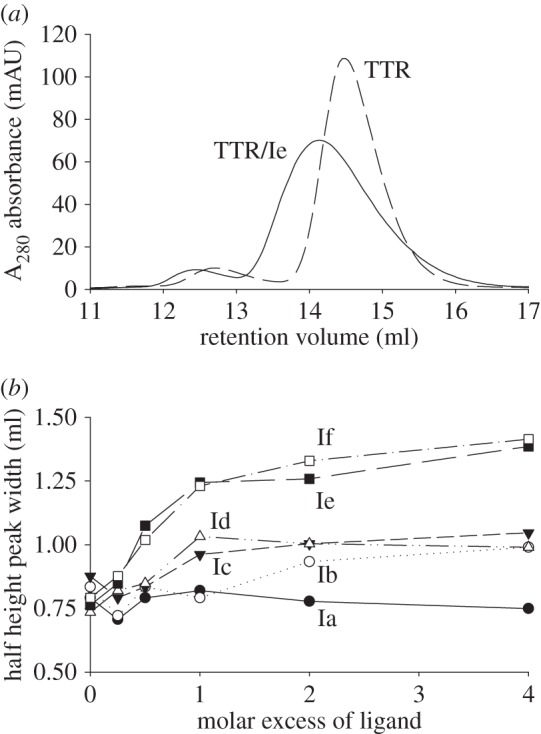
(*a*) The A_280_ UV absorbance gel filtration profiles of native TTR (dotted line) and TTR incubated with a fourfold excess of ligand Ie (Pro9, solid line). The ligand has caused an increase in peak volume and a concomitant decrease in retention volume consistent with the formation of a ligand octamer complex followed by dissociation on the column. (*b*) The effect of ligands on peak width is dose-dependent for all ligands except Ia (Pro5) where no octamer formation has occurred.

Gel filtration of native tetrameric TTR on Superdex 200 eluted with PBS yielded a major component eluting at 14.4 ± 0.2 ml and with a peak width at half height of 0.8 ml. There was also a minor peak, comprising about 5% of the total protein, at Ve 12.7 ml (figure 3*a*), produced by the naturally occurring dimerization of pairs of TTR tetramers [22]. Overnight incubation of TTR with up to a fourfold molar excess of the pentaproline ligand (Ia) had no effect on the gel filtration profile of the protein, with the main A_280_ component eluting at 14.5 ml with a peak width of 0.8 ml; there was, however, a fourfold increase in the A_330_/A_280_ UV absorbance ratio for the tetrameric complex at the highest ligand concentration, demonstrating that the ligand had been bound by TTR. In contrast, incubation of TTR with each of the remaining polyproline ligands (Ib–If, Pro6–10) caused a dose-dependent broadening of the TTR peak, with an increase in half-height peak width by up to 0.62 ml (figure 3*b*), together with a concomitant reduction in TTR elution volume. This was consistent with formation of crosslinked pairs of native tetrameric TTR molecules in the presence of excess ligand and their subsequent dissociation as free ligand was removed during gel filtration. The largest effect on peak width and retention time was observed with the Pro9 and Pro10 ligands, Ie and If. When the main A_280_-absorbing fractions from these samples were re-chromatographed on Superdex 200 each of the proteins eluted in the native position and with native half-height peak width (data not shown), showing that the complexes had completely dissociated when all the free ligand was removed by gel filtration. The formation of a complex of two TTR molecules by the Pro9 containing ligand Ie was confirmed by sedimentation ultracentrifugation in the presence of excess ligand, with appearance of a new species at 5.2S compared with native tetrameric TTR alone at 3.9S.

The presence and composition of ligand crosslinked TTR complexes was also investigated by nanoflow electrospray mass spectrometry. Under low-energy conditions designed for observation of non-covalent protein–protein interactions, native TTR populated four charge states (11+ to 14+) corresponding to the expected tetrameric assembly (56 500 ± 50 Da). After incubation with 0.5- to twofold molar equivalents of group I ligands there was dose-dependent appearance of TTR tetramers with one, and eventually two, bound ligand molecules occupying the thyroxine binding sites. With ligands Ib to Ie, low-intensity peaks (less than 10%) assigned to pairs of TTR tetramers with one to three bound ligand molecules were observed confirming the interpretation of the gel filtration findings. As these ligands did not generate stable octameric complexes with TTR, they were not studied further.

The polypiperidine ligands, IIa (Pip_3_.CO.Pip_3_) and IIb (Pip_4_.CO.Pip_1_), had effects similar to the group I ligands. There was some crosslinking that dissociated on gel filtration although the maximum peak widths produced by IIa and IIb, 1.6 and 2.1 ml, respectively, were considerably greater than obtained with any of the group I polyproline ligands suggesting that the polypiperidine complexes were more stable. Again, the complexes dissociated back to tetramer when subjected to a second gel filtration.

**Figure 4.**
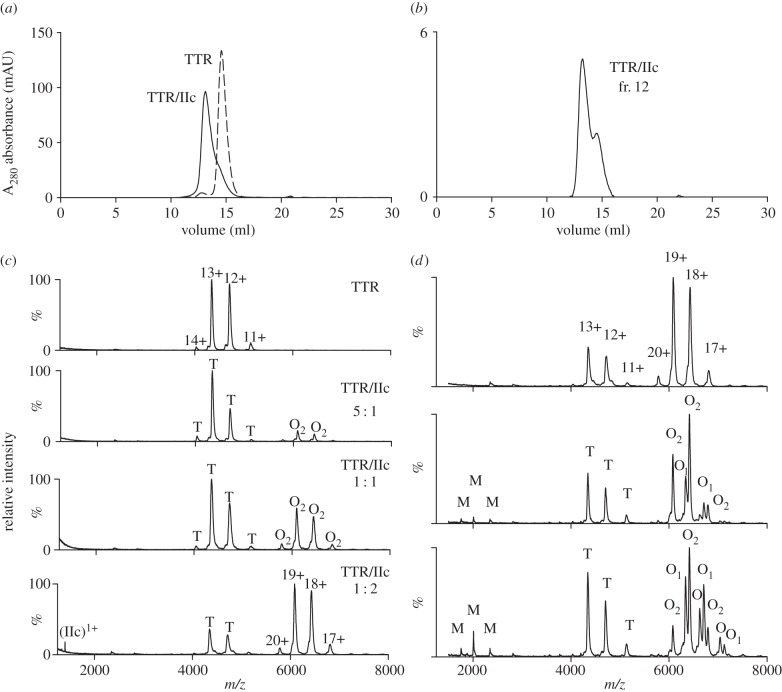
(*a*) Treatment of TTR with the polypiperidine ligand IIc generates an octameric complex which elutes on gel filtration with a retention volume of 12.8 ml and is separated from native tetrameric TTR at 14.6 ml. (*b*) The octamer (fraction 12) is stable to a second step of gel filtration. (*c*) Mass spectrometric examination of the complex of TTR with ligand IIc is consistent with the formation of a bis ligand octamer. Native apo TTR appears as the tetramer (T, charge states 11+ to 14+, 56 500 ± 50 Da). Addition of increasing levels of the ligand results in the dose dependent formation of an octameric species containing two ligands (O_2_, charge states 17+ to 20+, 115 715 ± 50 Da). (*d*) The TTR/IIc complex dissociates as the desolvation energy is increased (80, 100 and 120 V, top to bottom panels). The holo octameric TTR complex with two ligands bound (17+ to 20+, O_2_) dissociates to the octamer with one ligand (O_1_) and apo octamer (O) confirming stoichiometry of the TTR ligand complex formed. Release of monomers (M) also occurs under increasing desolvation energy. Mass spectra for the TTR/IId complex are shown in the electronic supplementary material, S1.

In contrast, ligands IIc (Pip_4_.CO.Pip_4_) and IId (Pip_5_.CO.Pip_5_) produced stable crosslinking with the complex eluting at 12.8 ml (figure 4*a*). Complex formation was dose-dependent, with over 90% of the TTR incorporated following 18 h incubation in the presence of a twofold molar excess of ligand, and these assemblies were stable to a second gel filtration step (figure 4*b*). Mass spectrometric analysis confirmed that treatment of TTR with ligands IIc or IId dose-dependently produced substantial amounts of a higher mass complex. Surprisingly, the molecular mass of approximately 116 000 Da was consistent with two TTR tetramers crosslinked by two ligand molecules (figure 4*c*) and this stoichiometry was confirmed under dissociating MS conditions (figure 4*d*). In contrast with the reversibly bound group I and group II ligands, no species were seen at normal operating cone voltages with either ligand bound by native tetrameric TTR molecules, or one or three ligand molecules bound by pairs of tetramers.

### 3.3. *In vivo* clearance studies

**Figure 5.**
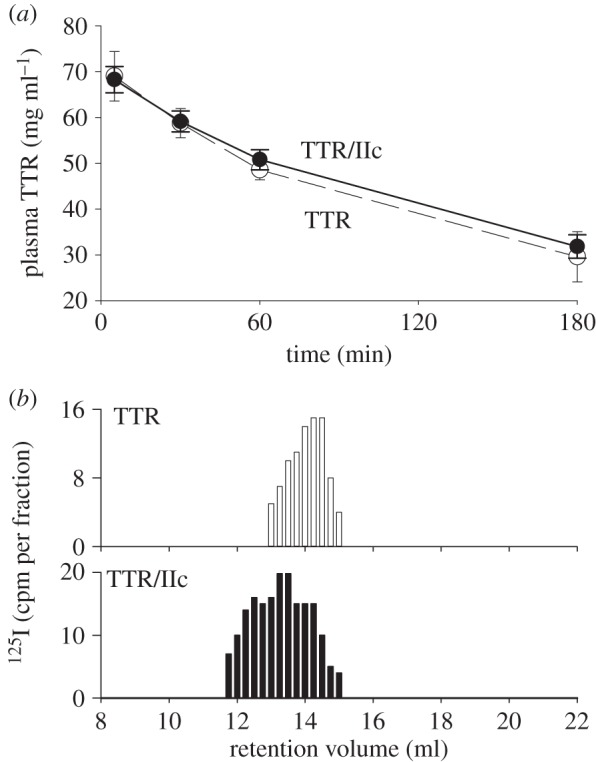
(*a*) There is no difference in the clearance of TTR and the stable TTR/IIc octamer in TTR knockout mice. (*b*) Analysis of the 60 min serum samples showed that radiolabelled octamer was still present in the circulation.

Native TTR was spiked with a trace of ^125^I-TTR and then incubated with a twofold excess of ligand IIc (Pip_4_.CO.Pip_4_). Although the A_280_ gel filtration profile confirmed more than 95% crosslinking of TTR protein only 60% of the ^125^I radiolabel was in the complex, indicating that oxidative radioiodination of TTR had partially inactivated the thyroxine binding sites. The tracer was thus not ideal for monitoring *in vivo* clearance of the TTR–ligand complex. Nevertheless, 200 µg of the preformed TTR–IIc complex containing ^125^I-TTR was injected intravenously into mice, and its clearance from the plasma was quantified both by radioactive counting and by specific immunoassay for human TTR. There was no change in the rate of clearance of the complex compared with control; the estimated half-lives were 82.2 and 68.7 min, respectively, both of which were within the range observed in our laboratory for clearance of native TTR in TTR knockout mice (mean (s.d.), 73.1 (37.2) min, *n* = 8; figure 5*a*). The 60 min serum samples from animals that had received control and ligand-treated TTR were fractionated by gel filtration and analysed for radioactivity. Native size, tetrameric ^125^I-TTR was present in the control serum but serum from the mice given the ligand-treated TTR contained a mixture of tetrameric TTR and dimers thereof (figure 5*b*). Because 40% of the radiolabelled ^125^I-TTR was not capable of ligand binding, these results demonstrate that the TTR–ligand complex is stable *in vivo* but is not cleared at an accelerated rate.

**Figure 6.**
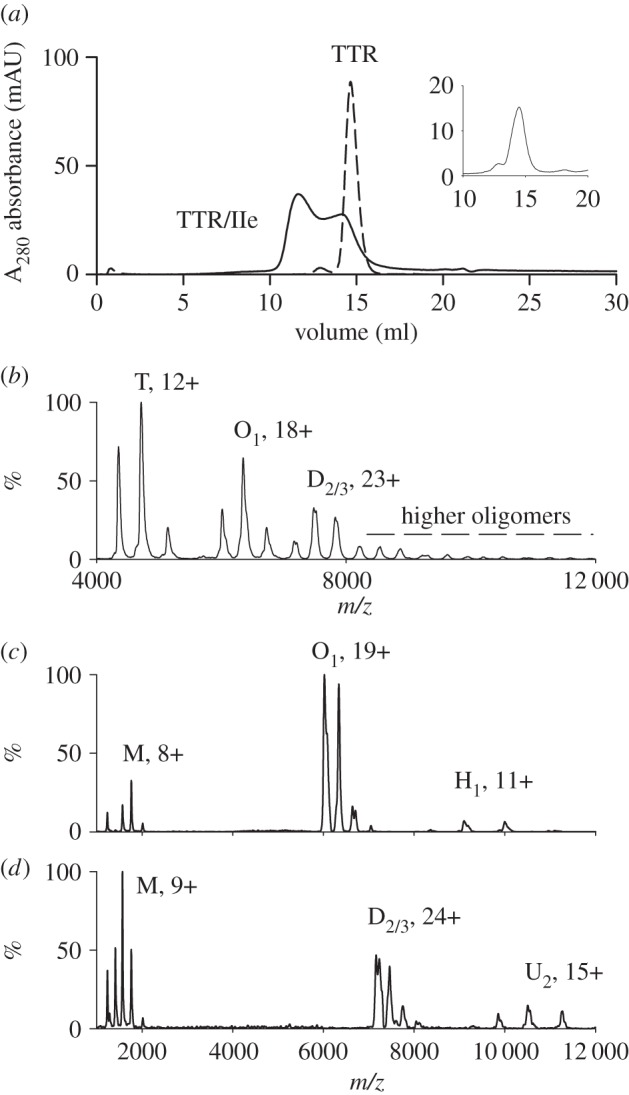
(*a*) The complex formed between ligand IIe and TTR contained an appreciable amount of a larger molecular mass species chromatographing on gel filtration as a dodecamer at approximately 11.0 ml (solid line) and separated from native TTR (dotted line). The complex was unstable, regenerating the tetramer (at 14.7 ml) together with a small amount of octamer (12.8 ml) on a second step of gel filtration (inset). (*b*) Nanoflow electrospray mass spectrum of TTR in the presence of 1 molar equivalent of ligand IIe. Under soft desolvation conditions, several charge state series are observed corresponding to apo tetrameric TTR (11+ to 13+, T), holo octameric TTR with one ligand bound (17+ to 19+, O_1_) and holo dodecameric TTR with two or three ligands bound (22+ to 24+, D_2/3_). Higher oligomeric species are also observed above 8000 *m*/*z*. (*c*) Tandem mass spectrum of the 19+ charge state of O_1_ shows the release of individual monomeric TTR (M) and the formation of ‘stripped complex’ H_1_ corresponding to heptameric TTR with one ligand bound. (*d*) Tandem mass spectrum of the 24+ charge state of D_2/3_ shows the release of individual monomeric TTR (M) and the formation of ‘stripped complex’ U_2_ corresponding to undecameric TTR with two ligands bound. Ion charges are shown for the most intense species in each ion series.

A number of other ligands with polypiperidine linkers were also synthesized, one of which, IIe, formed a higher molecular mass complex with TTR, possibly a dodecamer, eluting on gel filtration at approximately 11.0 ml (figure 6*a*). The complex was not stable in the absence of excess ligand, breaking down to yield the tetramer on further gel filtration. Spectrophotometric analysis of the relative absorbance at 330 nm for ligand and 280 nm for TTR protein demonstrated that the high-mass complexes did contain the ligand. Furthermore, mass spectrometric analysis identified species with two tetrameric TTR molecules and one or two ligand molecules, three TTRs with two or three ligands, and some higher mass species (figure 6*b*), presumably representing daisy chains of alternating TTR tetramers, each with their two known binding sites, crosslinked by bifunctional ligands. The assignments were confirmed by tandem MS analysis of the octamer containing one ligand which shows the release of individual monomeric TTR and the formation of a ‘stripped complex’ of heptameric TTR with one ligand bound. Similar analysis of dodecameric TTR with two ligands bound also shows release of individual monomeric TTR together with the formation of stripped undecameric TTR containing two ligands (figure 6*c,d*).

## 4. Discussion

**Figure 7.**
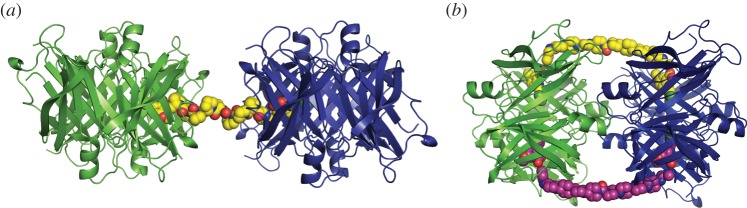
Models for the proposed TTR/Ie octameric complex (*a*) and the bracelet structure for TTR/IIc (*b*). The pictures were made using PyMOL Molecular Graphics System, version 0.99, Schrödinger, LLC.

The development of the hexanoyl bis(d-proline) drug, (*R*)-1-[6-[(*R*)-2-carboxy-pyrrolidin-1-yl]-6-oxo-hexanoyl]pyrrolidine-2-carboxylic acid (CPHPC), which rapidly and almost completely depletes its target protein, SAP, from the plasma for as long as the drug is administered, identified a novel pharmacological mechanism [12,13]. The essential feature is prompt hepatic clearance of the stable complex formed by two SAP molecules crosslinked by bifunctional ligand molecules. We speculated that this mechanism might operate with other pathogenic plasma protein targets if it was possible to design a suitably specific and avidly bound crosslinking ligand. Each native homotetrameric TTR molecule has two identical thyroxine binding pockets and the work of Purkey *et al.* [21] had identified (3,5-dichlorophenylamino)benzoic acid as a specific high affinity ligand bound in this site by TTR within the milieu of whole serum. It is thus a suitable starting point for construction of the required crosslinking structure. In our original effort to create such a compound, we attached the ligand head groups in an orientation that serendipitously enabled the molecule to enter one thyroxine binding site and then traverse the channel through the centre of the TTR molecule, so that both binding sites were occupied with the linker threaded internally through the protein [16]. Here, we therefore attached the head groups in the opposite orientation, to avoid such threading and to enable crosslinking of different TTR molecules to take place, and used linkers with lengths of 20–50 Å, sufficient, based on our modelling studies, to reach between two adjacent TTR tetramers (figure 7).

Our approach, of enhancing clearance of the amyloid precursor, differs from the TTR stabilization method which underlies the mode of action of the drugs currently in clinical use, tafamidis (Vyndaqel) and diflunisal [23–26]. While some degree of stabilization of the native TTR tetramer may also be conferred by our bifunctional ligands, the major beneficial effect would arise through depletion of plasma TTR rather than stabilization of the native tetramer. This may be important since recent work from our laboratory indicates that a mechano-enzymatic mechanism plays a prominent role in TTR amyloid fibrillogenesis [27] and that TTR tetramer stabilization alone may be insufficient to block amyloid formation if it does not also protect against the key proteolytic cleavage.

The rapid plasma clearance of the heterogeneous, mostly high mass, aggregates of TTR produced by covalent crosslinking with EDC is unsurprising and entirely as expected, because even trivial structural alteration of many plasma proteins, as for example after oxidative trace radioiodination or other inadvertent denaturation during isolation, has long been known to promote accelerated clearance by the liver and spleen. The major uptake of chemically aggregated TTR in the spleen is nonetheless of interest in view of the propensity of TTR and other types of amyloid to deposit in the spleen but we have no evidence that our model has any pathophysiological relevance in this context. We used EDC-TTR only to confirm that TTR aggregates would be cleared from the circulation more rapidly than single native TTR molecules and to show that we could detect the difference, before going on to test the effect of our crosslinking ligands.

Rigid polyproline chains, Pro5–Pro10 in compounds Ia–If respectively, were initially used as linkers, and other than Ia (Pro5) each ligand was capable of crosslinking TTR consistent with a minimum inter-tetramer distance of approximately 30 Å in the TTR–ligand–TTR complex (figure 7*a*). However, the complexes were unstable in the absence of excess free ligand. Nevertheless, mass spectrometric analysis of mixtures of TTR with excess ligands identified complexes of TTR with one or two ligand molecules bound and a low abundance of pairs of TTR molecules with one, two and three bound ligand molecules. Thus, if the stability problem could be overcome, both pairs and higher oligomers of TTR could potentially be produced.

In contrast to the rigid polyproline linker compounds, two of the compounds with flexible polypiperidine linkers, IIc and IId, produced dose-dependent stable crosslinking of pairs of TTR molecules. These complexes each contained two ligand molecules consistent with a bracelet structure in which the linkers encircle the globular TTR protein assembly (figure 7*b*). With a small rotation of the TTR tetramers, the approximately 40 Å length of the polypiperidine linker in IIc is compatible with the head groups being engaged in the binding pockets, whereas the linker chain emerges through a furrow normal to the TTR head group axis. Acidic residues flanking the furrow could additionally stabilize the complex in comparison with the ‘daisy chain’ crosslinking configuration provided by the rigid linear polyPro compounds.

Although the TTR–IIc complex was stable *in vivo* as well as *in vitro*, it was not cleared from the circulation at an accelerated rate. This may reflect the fact that a substantial proportion of TTR normally circulates in a stable complex with retinol binding protein and the modestly increased size of the TTR–IIc complex is insufficient to trigger enhanced clearance. The modelled radius of gyration is only 29.96 Å compared with 22.67 Å for the native TTR tetramer. Unlike SAP, no simple crosslinking of pairs of TTR molecules will lead to TTR depletion from the plasma. Larger oligomers, as produced by covalent crosslinking with EDC, were swiftly cleared, and in theory, therefore, higher-order oligomers in the daisy chain configuration produced by rigid linker ligands should be effectively cleared. Here, we observed the complexes of three TTR molecules with compound IIe, but potent ligands of this type will require head groups that are specifically bound by TTR with much greater affinity than any we have yet identified or others have reported.

## Supplementary Material

Bifunctional cross linking ligands for transthyretin: Synthesis of ligands

## Supplementary Material

Datasets for figures 2-6 and S1
