# Supplementary material for: Bifunctional crosslinking ligands for transthyretin
Source: Open Biol. 2015 Sep 23;5(9):150105. doi: 10.1098/rsob.150105 (PMC4593668; doi:10.1098/rsob.150105)
Supplement: Bifunctional cross linking ligands for transthyretin: Synthesis of ligands [file rsob150105supp1.pdf]

Electronic Supplementary Material. Bifunctional cross linking ligands for transthyretin, Mangione *et al.*

A. Supplementary figure S1

A. Mass spectrometric examination of the complex of TTR with ligand IId is consistent with the formation of a bis-ligand octamer also observed with ligand IIc. Native apo TTR appears as the tetramer (T, charge states 11+ to 13+, 56,500 ± 50 Da). Addition of increasing levels of the ligand results in the dose-dependent formation of an octameric species containing two ligands (O_2, charge states 17+ to 19+, 116,010 ± 50 Da).
B. The TTR/IId complex dissociates as the desolvation energy is increased (top to bottom panels, cone voltages 80 – 100 – 120 V). The holo octameric TTR complex with 2 ligands bound (17+ to 19+, O_2) dissociates to the octamer with 1 ligand (O_1) and apo octamer (O) confirming stoichiometry of the TTR ligand complex formed. Monomer (M) formation and ligand dissociation occur at the same time.

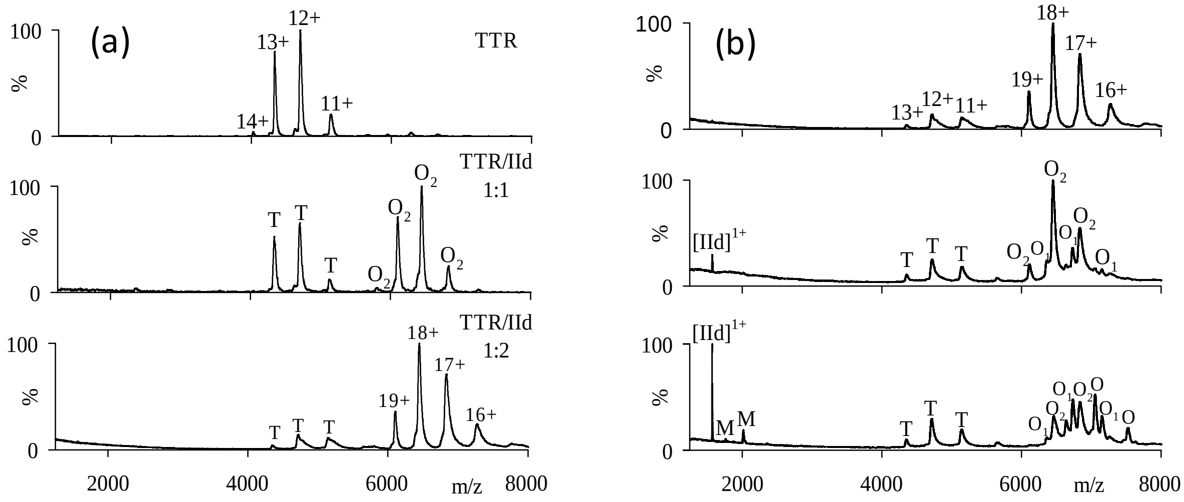

B. Synthesis of ligands

2-(3,5-Dichloro-phenylamino)-5-methoxy-benzoic acid methyl ester

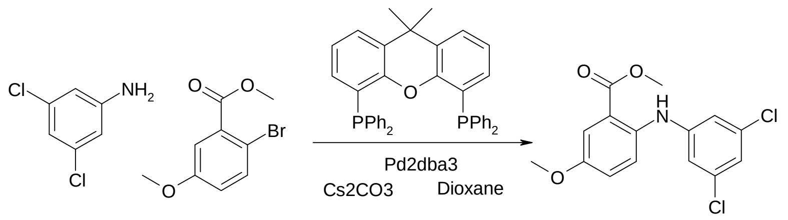

3,5-Dichloroaniline (1 g, 6.17 mmol) was dissolved in dioxane (75 mL) and 4,5-bis(diphenylphosphino)-9,9-dimethylxanthene (595 mg, 1.028 mmol), tris(dibenzylideneacetone)dipalladium(0) (470 mg, 0.514 mmol), cesium carbonate (5.02 g, 15.42 mmol) and methyl 2-bromo-5-methoxybenzoate (0.83 mL, 5.14 mmol) were added. The reaction was degassed with vacuum/nitrogen x 3, then heated to reflux and stirred under nitrogen for 18 h. The reaction was allowed to cool to room temperature, then concentrated *in vacuo*. The mixture was then partitioned between water and EtOAc. The aqueous phase was separated and extracted 3 x EtOAc. The combined organics were washed with brine, dried over $MgSO_4$, filtered and concentrated *in vacuo*. The orange oil was dissolved in DCM and pre-adsorbed onto silica gel, then purified by flash column chromatography on 200 ml silica gel with 2-8% EtOAc in *i*-hexanes to afford the title compound (1.15 g) as a yellow solid. ESMS $[M+H]^+$ = 326.02, ^1H NMR (300 MHz, $CDCl_3$) δ: 9.07 (1H, s), 7.50 (1H, d, *J* = 2.87 Hz), 7.33 (1H, d, *J* = 9.28 Hz), 7.08 (1H, dd, *J* = 2.87, 9.28 Hz), 7.04 (2H, s), 6.94 (1H, s), 3.93 (3H, s), 3.83 (3H, s).

2-(3,5-Dichloro-phenylamino)-5-hydroxy-benzoic acid methyl ester

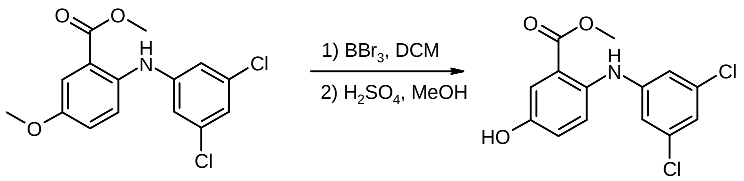

To an anhydrous solution of 2-(3,5-dichloro-phenylamino)-5-methoxy-benzoic acid methyl ester (6 g, 18.4 mmol) in DCM (50 ml) at –5°C, was added BBr_3 (5.2 ml, 55.2 mmol) dropwise under nitrogen. The resulting mixture was left to stir at –5°C for 30 mins. TLC analysis showed no starting material present. Water was carefully added and the resulting mixture was extracted with DCM. The combined organic layer was washed with brine, dried over Na_2SO_4 and filtered. The filtrate was evaporated under reduced pressure to give a crude oil. This was left under vacuum to dry and then taken into the next step.

The crude oil was taken up in MeOH (100 ml) and 6 drops of conc. sulphuric acid was added. The solution was heated under reflux for 8 h. The solution was then evaporated to give colourless oil. This was partitioned between EtOAc and water. The aqueous layer extracted with additional EtOAc. The combined organic layer washed with brine, dried over sodium

sulfate and filtered. The filtrate was evaporated under reduced pressure followed by drying under vacuum to give the title compound as yellow solid (5.8 g). ESMS $[M+H]^+$ = 310.05, ^1H NMR (300 MHz, DMSO-D6) δ: 9.61 (1H, s), 8.45 (1H, s), 7.27 (1H, d, *J* = 2.85 Hz), 7.24 (1H, d, *J* = 8.64 Hz), 7.01 (1H, dd, *J* = 2.85, 8.64 Hz), 6.87 (3H, s), 3.77 (3H, s).

5-(3-Tert-butoxycarbonylamino-propoxy)-2-(3,5-dichloro-phenylamino)-benzoic acid methyl ester

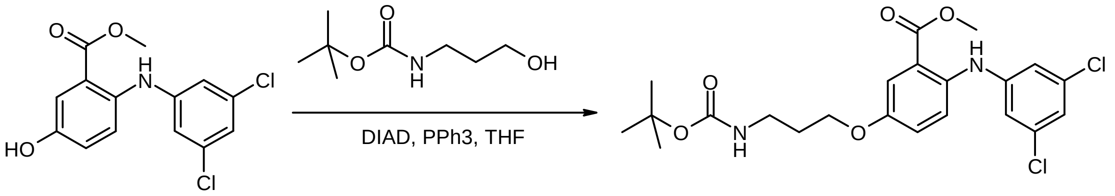

Under N_2, 2-(3,5-dichloro-phenylamino)-5-hydroxy-benzoic acid methyl ester (2 g, 6.4 mmol) was dissolved in THF (25 ml). *t*-Butyl-N-(3-hydroxypropyl)carbamate (1.31 ml, 7.68 mmol) and triphenylphosphine (2.52 g, 9.6 mmol) were then added, followed by DIAD (1.51 ml, 7.7 mmol). The reaction was allowed to stir overnight, then quenched by addition of $NaHCO_3$ (sat. aq.), extracted x 3 with ethyl acetate, the combined organics washed with brine, dried over Na_2SO_4, filtered and concentrated *in vacuo*. The residue was purified by flash column chromatography with 10-20% EtOAc/*i*-hexanes to afford 2.7 g of yellow foam. LCMS showed 86% of the title compound ESMS $[M+H]^+$ = 469.12, 14% recovered phenol. The mixture was taken into the next step.

5-(3-Amino-propoxy)-2-(3,5-dichloro-phenylamino)-benzoic acid methyl ester

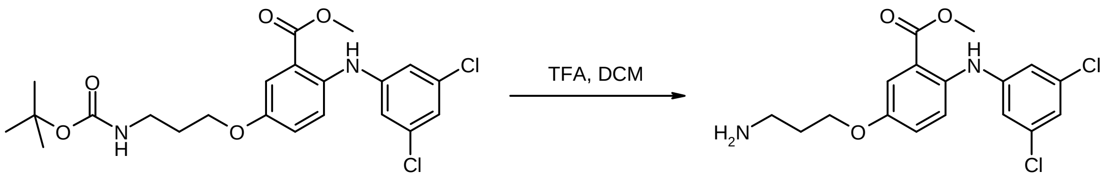

The mixture from the previous experiment (2.7 g) was dissolved in DCM (23 ml) and TFA added (5.7 ml). The reaction was allowed to stir at RT for 45 mins. The mixture was then concentrated *in vacuo*, redissolved in DCM and quenched with $NaHCO_3$ (sat. aq.). 0.880 NH_3 was then added to the mixture until complete dissolution occurred. The aqueous was then extracted 3 x DCM, the combined organics dried over Na_2SO_4, filtered and concentrated *in vacuo*. The residue was purified by flash column chromatography 10-20% MeOH/DCM. The

title compound (1.23 g) was obtained as a yellow solid. ESMS $[M+H]^+$ = 369.07. ^{1}H NMR (300 MHz, $CDCl_3$) 9.06 (1H, s), 7.51 (1H, d, *J* = 2.88 Hz), 7.32 (1H, d, *J* = 9.06 Hz), 7.07 (1H, dd, *J* = 2.88, 9.06 Hz), 7.04 (1H, s), 7.03 (1H, s), 6.93 (t, *J* = 1.77 Hz), 4.07 (2H, t, *J* = 6.21 Hz), 2.94 (2H, t, *J* = 6.84 Hz), 1.95 (2H, m).

The higher running spot was also collected from the column to give a yellow solid (457 mg), which was the starting phenol. ESMS $[M+H]^+$ = 310.05, ^{1}H NMR (300 MHz, DMSO-D6) δ: 9.61 (1H, s), 8.45 (1H, s), 7.27 (1H, d, *J* = 2.85 Hz), 7.24 (1H, d, *J* = 8.64 Hz), 7.01 (1H, dd, *J* = 2.85, 8.64 Hz), 6.87 (3H, s), 3.77 (3H, s).

4-(1,4-Dioxa-8-aza-spiro[4.5]dec-8-yl)-[1,4']bipiperidinyl-1'-carboxylic acid benzyl ester

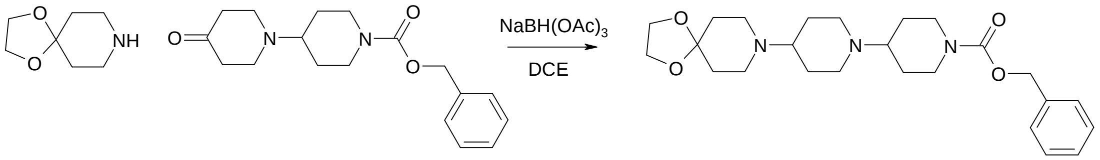

4-Oxo-[1,4']bipiperidinyl-1'-carboxylic acid benzyl ester (*Angew Chem Int Ed Eng*. **2006**, *45(4),* 588, 4 g, 12.64 mmol) was dissolved in DCE (60 mL) and 1,4-dioxa-8-spiro[4,5]decane (1.94 mL, 15.2 mmol) and sodium triacetoxyborohydride (5.36 g, 25.28 mmol) were added. The reaction was stirred under N_2 overnight. The reaction was then quenched with $NaHCO_3$ (sat. aq.), extracted 3 x DCM, the combined organics were washed with brine, dried over Na_2SO_4, filtered and concentrated *in vacuo*. The colourless solid was then triturated with diethyl ether, and collected by filtration, washing with diethyl ether to afford the title compound (4.160 g) as a colourless solid. ESMS $[M+H]^+$ = 444. ^{1}H NMR (300 MHz, $CDCl_3$) δ: 7.38-7.30 (5H, m), 5.13 (2H, s), 4.24 (2H, bd, *J* = 8.4 Hz), 3.96 (4H, s), 2.97 (2H, d, *J* = 11.3 Hz), 2.78 (2H, app t, *J* = 12.1 Hz), 2.66-2.63 (4H, m), 2.47-2.31 (2H, m), 2.17 (2H, app t, *J* = 11.0 Hz), 1.82-1.74 (8H, m), 1.63-1.38 (4H, m).

4-Oxo-[1,4';1',4'']terpiperidine-1''-carboxylic acid benzyl ester

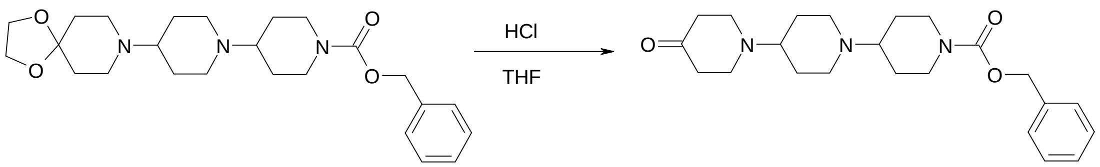

The 4-(1,4-dioxa-8-aza-spiro[4.5]dec-8-yl)-[1,4']bipiperidinyl-1'-carboxylic acid benzyl ester (4.15 g, 9.36 mmol) was dissolved in THF and 5N HCl (40 ml) was added. The reaction was

heated to 100°C and allowed to stir at this temperature for 3 h. The mixture was then cooled to 0°C then quenched with NaOH (5N, aq.) until pH 10, as judged by universal pH paper. The mixture was extracted 3 x EtOAc, dried over Na_2SO_4, and concentrated *in vacuo* to afford the title compound (4.6 g) as a pale yellow oil. ^{1}H NMR showed that the product still contained ethylene glycol. ESMS $[M+H]^+$ = 444. ^{1}H NMR (300 MHz, $CDCl_3$) δ: 7.39-7.30 (5H, m), 5.13 (2H, s), 4.24 (2H, m), 2.97 (2H, d, *J* = 11.5 Hz), 2.88-2.74 (6H, m), 2.50-2.41 (5H, m), 2.20 (2H, app t, *J* = 11.3 Hz), 1.95-1.68 (5H, m), 1.64-1.40 (4H, m).

4-Oxo-[1,4';1',4'']terpiperidine-1''-carboxylic acid tert-butyl ester

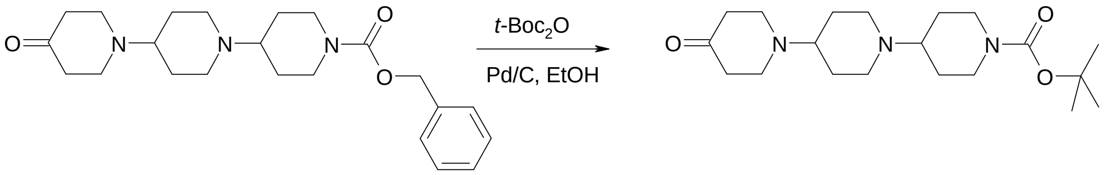

4-(1,4-Dioxa-8-aza-spiro[4.5]dec-8-yl)-[1,4']bipiperidinyl-1'-carboxylic acid benzyl ester (3.74 g, 9.36 mmol) was dissolved in EtOH (120 mL) and $tBoc_2O$ (2.66 g, 12.16 mmol) was added. The mixture was flushed with vacuum and N_2 x 3, then the Pd/C was added. The mixture was then flushed with vacuum and H_2 x 3, then allowed to stir under a balloon of H_2 overnight. The mixture was then flushed with vacuum and N_2 x 3, and filtered through hyflo, washing with MeOH. The mixture was concentrated *in vacuo*, then purified by flash column chromatography with 5% (10% 0.880 NH_3 in MeOH)/DCM to afford the title compound (2.90 g) as a colourless oil which solidified upon drying under high vacuum. ESMS $[M+H]^+$ = 366. ^{1}H NMR (300 MHz, $CDCl_3$) δ: 4.16 (2H, bd, *J* = 11.3 Hz), 3.00 (2H, d, *J* = 11.5 Hz), 2.86 (4H, app t, *J* = 6.0 Hz), 2.73-2.65 (2H, m), 2.45 (6H, app t, *J* = 6.2 Hz), 2.20 (2H, app t, *J* = 10.4 Hz), 1.84-1.76 (4H, m), 1.66-1.25 (13H, m).

4-{3-[4-(3,5-Dichloro-phenylamino)-3-methoxycarbonyl-phenoxy]-propylamino} [1,4';1',4'']terpiperidine-1''-carboxylic acid tert-butyl ester

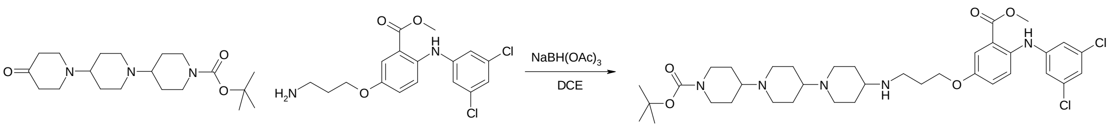

4-Oxo-[1,4';1',4"]terpiperidine-1"-carboxylic acid tert-butyl ester (772 mg, 2.11 mmol) and 5-(3-amino-propoxy)-2-(3,5-dichloro-phenylamino)-benzoic acid methyl ester (600 mg, 1.62 mmol) were dissolved in DCE (12 mL) and sodium triacetoxyborohydride (688 mg, 3.24 mmol) was added. The mixture was stirred under N_2 for 3 h. The reaction was quenched by the addition of $NaHCO_3$ (sat. aq.), extracted 3 x DCM and the combined organics washed with brine, dried over Na_2SO_4, filtered and concentrated *in vacuo*. The mixture was purified by silica gel chromatography eluting with a gradient of 5-10% (10% 0.880 NH_3 in MeOH)/DCM to afford a pale yellow foam. The foam was dried under high vacuum to afford the title compound (1.069 g) as a pale yellow foam. ESMS $[M+H]^+$ = 718.48. ^{1}H NMR (300 MHz, $CDCl_3$) δ: 9.06 (1H, s), 7.49 (1H, d, *J* = 3.1 Hz), 7.31 (1H, d, *J* = 9.1 Hz), 7.06 (1H, dd, *J* = 9.1, 3.1 Hz), 7.04-7.03 (2H, m), 6.94 (1H, app t, *J* = 1.3 Hz), 4.14 (2H, bd, *J* = 10.4 Hz), 4.05 (2H, t, *J* = 6.0 Hz), 3.91 (3H, s), 2.97 (2H, d, *J* = 11.5 Hz), 2.91-2.81 (4H, m), 2.69 (2H, app t, *J* = 11.9 Hz), 2.49-2.32 (3H, m), 2.28-2.12 (4H, m), 2.02-1.90 (4H, m), 1.80-1.77 (4H, m), 1.61-1.25 (16H, m).

2-(3,5-Dichloro-phenylamino)-5-[3-([1,4';1',4'']terpiperidin-4-ylamino)-propoxy]-benzoic acid methyl ester

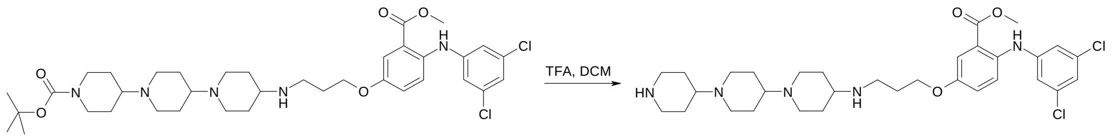

4-{3-[4-(3,5-Dichloro-phenylamino)-3-methoxycarbonyl-phenoxy]-propylamino}[1,4';1',4"] terpiperidine-1"-carboxylic acid tert-butyl ester (966.9 mg, 1.35 mmol) was dissolved in DCM (12 mL) and trifluoroacetic acid (3 mL) added dropwise. The reaction was stirred under N_2 for 45 min, then concentrated *in vacuo*. The residue was partitioned between DCM and $NaHCO_3$ (sat. aq.) and 0.880 NH_3 was added until the solid TFA salt went into solution. The aqueous phase was then extracted 3 x DCM, the combined organics were washed with a 1:1 mixture of 0.880 NH_3 and H_2O, and then with brine. The organics were dried over Na_2SO_4, filtered, concentrated *in vacuo* and dried under high vacuum to afford the title compound (797 mg) as a yellow foam. ESMS $[M+H]^+$ = 618.43. ^{1}H NMR (300 MHz, $CDCl_3$) δ: 9.07 (1H, s), 7.48 (1H, d, *J* = 3.1 Hz), 7.31 (1H, d, *J* = 9.1 Hz), 7.06 (1H, dd, *J* = 9.1, 3.1 Hz), 6.93 (1H, app t, *J* = 1.8 Hz), 4.04 (2H, t, *J* = 6.0 Hz), 3.91 (3H, s), 3.15 (2H, d, *J* = 12.4 Hz), 3.00 (2H, d, *J* = 11.5 Hz), 2.91-2.81 (4H, m) 2.58 (2H, app t, *J* = 11.3 Hz), 2.49-2.14 (7H, m), 2.00-1.91 (4H, m), 1.82-1.78 (5H, m) 1.62-1.25 (9H, m).

Bis-(1,4-dioxa-8-aza-spiro[4.5]dec-8-yl)-methanone

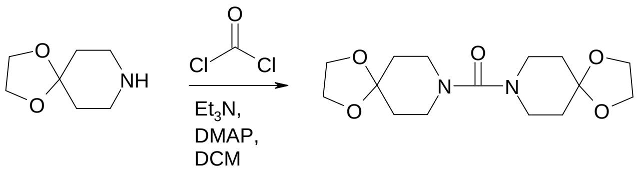

1,4-Dioxa-8-azaspiro[4,5]decane (3.6 mL, 2.8 mmol) was dissolved in DCM (80 mL) and triethylamine (7.8 mL, 5.6 mmol) and phosgene (10% in toluene, 7.28 mL, 1.4 mmol) added, followed by 4-DMAP (80 mg, 0.65 mmol). The reaction was allowed to stir under N_2 for 1 h. An additional 0.1 eq. phosgene was then added (1.46 mL) and the reaction was shown to be complete by TLC. The reaction was quenched with water, extracted 3 x EtOAc, washed with 2N HCl, then dried over Na_2SO_4, filtered and concentrated *in vacuo* to give the title compound (3.81 g) as colourless crystals. ESMS $[M+H]^+$ = 313.18. ^1H NMR (300 MHz, $CDCl_3$) δ: 3.97 (8H, s), 3.34 (8H, app t, *J* = 5.7 Hz), 1.71 (8H, app t, *J* = 5.7 Hz).

Bis-(4-oxapiperidyl)-methanone

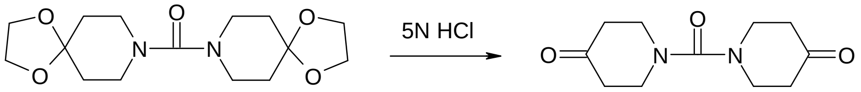

Bis-(1,4-dioxa-8-aza-spiro[4.5]dec-8-yl)-methanone (3.81 g, 12.2 mmol) was dissolved in 5N HCl and allowed to stir for 24 h. The reaction was then cooled to 0°C and quenched with 5N NaOH until pH 9, as judged by universal pH paper. The mixture was then extracted 3 x EtOAc, dried over Na_2SO_4, filtered and concentrated *in vacuo* to afford a colourless solid. The solid was triturated with 1:1 diethyl ether and *i*-hexanes and filtered off, then dried under high vacuum to afford the title compound (1.40 g) as a colourless solid. ESMS $[M+H]^+$ = 225. ^1H NMR (300 MHz, $CDCl_3$) δ: 3.63 (8H, app t, *J* = 6.2 Hz), 2.54 (8H, app t, *J* = 6.2 Hz). ^{13}C NMR (300 MHz, $CDCl_3$) δ: 207.6, 163.6, 46.8, 41.6.

IIc Dimethyl ester

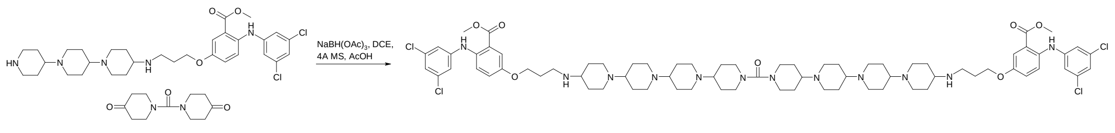

Bis-(4-oxapiperidyl)-methanone (32 mg, 0.144 mmol) and 2-(3,5-dichloro-phenylamino)-5-[3-([1,4';1',4"]terpiperidin-4-ylamino)-propoxy]-benzoic acid methyl ester (179 mg, 0.289 mmol) were dissolved in DCE (2.5 mL) and 4 Å molecular sieves (100 mg) and acetic acid (1 drop) were added. After stirring for 2 h under N_2, sodium triacetoxyborohydride was added

(245 mg, 0.578 mmol). The reaction was stirred under N_2 for 72 h. The reaction was quenched by addition of $NaHCO_3$, and extracted 3 x DCM. The combined organics were washed with brine and dried over Na_2SO_4, filtered and concentrated *in vacuo* to afford a yellow oil. A precipitate was still visible in the aqueous. This was collected by filtration, and LCMS showed it to be the title compound (16 mg), which was a yellow solid.

The yellow oil was purified by flash column chromatography 10-16% (10% 0.880 NH_3 in MeOH)/DCM to afford the title compound (38 mg) as a yellow foam. ESMS $[M+H]^+$ = 1429.75. ^1H NMR (300 MHz, $CDCl_3$) δ: 9.06 (2H, s), 7.48 (2H, d, *J* = 2.9 Hz), 7.35 (2H, d, *J* = 9.1 Hz), 7.05 (2H, dd, d, *J* = 2.9, 9.1 Hz), 7.03-7.02 (4H, m), 6.92 (2H, app t, *J* = 1.8 Hz), 4.04 (4H, t, *J* = 6.0 Hz), 3.90 (6H, s), 3.71 (4H, *J* = 12.6 Hz), 3.01-2.92 (12H, m), 2.85 (4H, t, *J* = 6.8 Hz), 2.72 (4H, t, *J* = 12.2 Hz), 2.54-2.13 (16H, m), 2.00-1.96 (8H m), 1.81-1.79 (10H, m), 1.66-1.35 (16H, m), 1.27-1.25 (6H, m), 0.89-0.84 (2H).

IIc

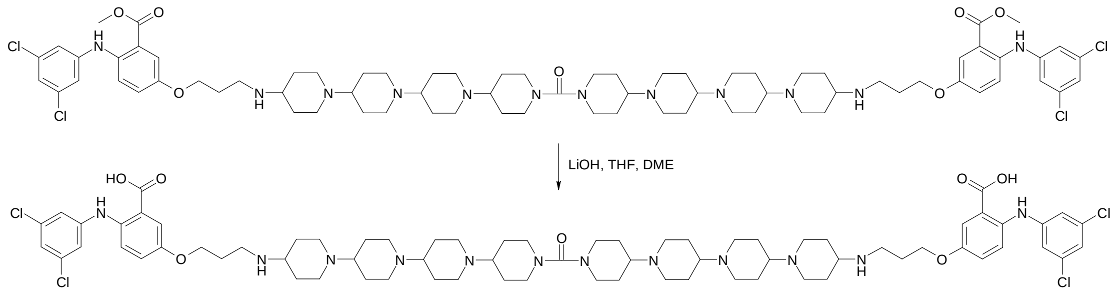

IIc dimethyl ester (39 mg, 0.0273 mmol) was suspended in THF (1.2 mL). The mixture was heated to aid solubility, however the compound did not dissolve. After cooling to RT, LiOH (0.5 M, 0.4 ml) was added and the mixture stirred overnight under N_2 at room temperature. LCMS showed only starting material. DME (2 ml) was added, and after stirring for 1 h at room temperature the reaction was heated to 45°C for 4 h, then stirred overnight at room temperature. LCMS showed completion. The fine precipitate was filtered off, and dried under high vacuum. ESMS $[M+H]^+$ = 1401.68. ^1H NMR (300 MHz, CD_3OD) δ: 7.56 (2H, d, *J* = 3.1 Hz), 7.25 (2H, d, *J* = 8.6 Hz), 6.98-6.93 (6H, m), 6.78 (2H, t, *J* = 1.8 Hz), 4.08 (4H, t, *J* = 6.0 Hz), 3.76-3.71 (6H, m), 3.04 (8H, d, *J* = 10.6 Hz), 2.95 (4H, d, *J* = 10.4 Hz), 2.84-2.75 (8H, m), 2.49-2.44 (4H, m), 2.29-2.13 (16H, m), 2.00-1.86 (20H, m), 1.59-1.30 (16H, m).

4-Oxo-[1,4']bipiperidinyl-1'-carboxylic acid tert-butyl ester

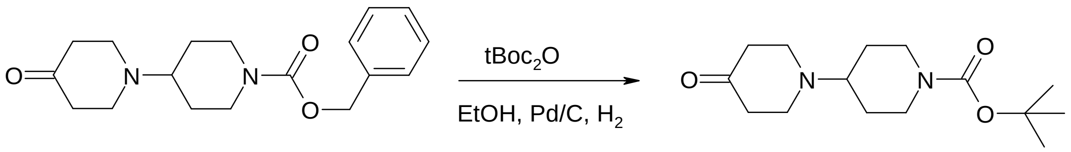

4-Oxo-[1,4']bipiperidinyl-1'-carboxylic acid benzyl ester (1 g, 3.16 mmol) was dissolved in ethanol (30 mL) and di-t-butyl dicarbonate (828 mg, 3.79 mmol) was added. The mixture was flushed three times with vacuum and H_2, the reaction was then allowed to stir under hydrogen for 16 h. The mixture was filtered through hyflo, washed with methanol and then concentrated *in vacuo*. The mixture was purified by flash column chromatography using 2% (10% 0.880 NH_3 in MeOH)/dichloromethane to afford the title compound (731 mg) as a colourless oil. ESMS $[M+MeOH+H]^+$ = 315.22. ^{1}H NMR (300 MHz, $CDCl_3$) δ: 4.28-4.10 (2H, m), 3.51 (2H, bs), 2.89-2.82 (2H, m), 2.80-2.56 (2H, m), 2.49-2.41 (2H, m), 1.85-1.76 (3H, m), 1.54-1.40 (11H, m).

4-{3-[4-(3,5-Dichloro-phenylamino)-3-methoxycarbonyl-phenoxy]-propylamino}-[1,4']bipiperidinyl-1'-carboxylic acid tert-butyl ester

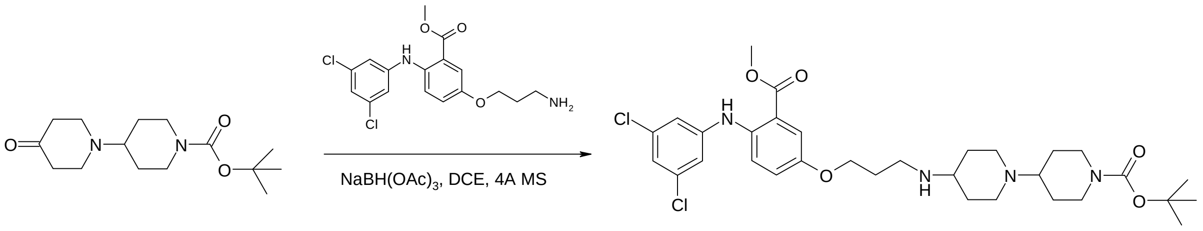

Prepared by the same procedure as 4-{3-[4-(3,5-dichloro-phenylamino)-3-methoxycarbonyl-phenoxy]-propylamino} [1,4';1',4"]terpiperidine-1"-carboxylic acid tert-butyl ester but using 4-oxo-[1,4']bipiperidinyl-1'-carboxylic acid tert-butyl ester (397 mg) instead of 4-oxo-[1,4';1',4"]terpiperidine-1"-carboxylic acid tert-butyl ester. The title compound (680 mg) was obtained as a yellow foam. ESMS $[M+H]^+$ = 635.43. ^{1}H NMR (300 MHz, $CDCl_3$) δ: 9.04 (1H, s), 7.46 (1H, d, *J* = 3.0 Hz), 7.28 (1H, d, *J* = 9.0 Hz), 7.03 (1H, dd, *J* = 9.0, 3.0 Hz), 6.99 (2H, d, *J* = 1.6 Hz), 6.88 (1H, t, *J* = 1.7 Hz), 4.20-4.06 (2H, m), 4.03 (2H, dd, *J* = 6.0, 6.0 Hz), 3.88 (3H, s), 2.91-2.76 (4H, m), 2.64 (2H, dd, *J* = 12.3, 12.3 Hz), 2.50-2.32 (2H, m), 2.19 (2H, dd, *J* = 11.2, 11.2 Hz), 1.99-1.84 (4H, m), 1.80-1.70 (2H, m), 1.48-1.20 (14H, m).

5-[3-([1,4']Bipiperidinyl-4-ylamino)-propoxy]-2-(3,5-dichloro phenylamino)benzoic acid methyl ester

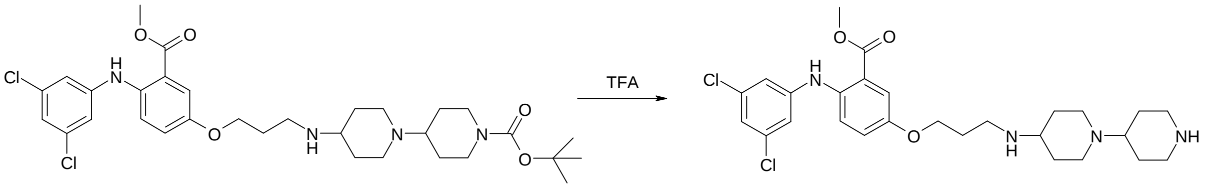

Prepared by the same procedure as 2-(3,5-dichloro-phenylamino)-5-[3-([1,4';1',4"] terpiperidin-4-ylamino)-propoxy]-benzoic acid methyl ester but using 4-{3-[4-(3,5-dichloro-phenylamino)-3-methoxycarbonyl-phenoxy]-propylamino}-[1,4']bipiperidinyl-1'-carboxylic acid tert-butyl ester (680 mg) instead of 4-{3-[4-(3,5-dichloro-phenylamino)-3-methoxycarbonyl-phenoxy]-propylamino} [1,4';1',4"]terpiperidine-1"-carboxylic acid tert-butyl ester. The title compound (572 mg) was obtained as a yellow oil. ESMS $[M+H]^+$ = 535.18. ^{1}H NMR (300 MHz, $CDCl_3$) δ: 9.03 (1H, s), 7.45 (1H, d, *J* = 3.0 Hz), 7.27 (1H, d, *J* = 9.0 Hz), 7.03 (1H, dd, *J* = 9.0, 3.0 Hz), 6.990 (2H, d, *J* = 1.6 Hz), 6.88 Hz (1H, t, *J* = 1.8 Hz), 4.01 (2H, dd, *J* = 6.1, 6.1 Hz), 3.88 (3H, s), 3.16-3.15 (2H, m), 2.93-2.75 (4H, m), 2.61-2.49 (2H, m), 2.49-2.28 (2H, m), 2.26-2.14 (2H, m), 1.99-1.84 (4H, m), 1.82-1.73 (2H, m), 1.47-1.18 (6H, m).

IIa Methyl Ester

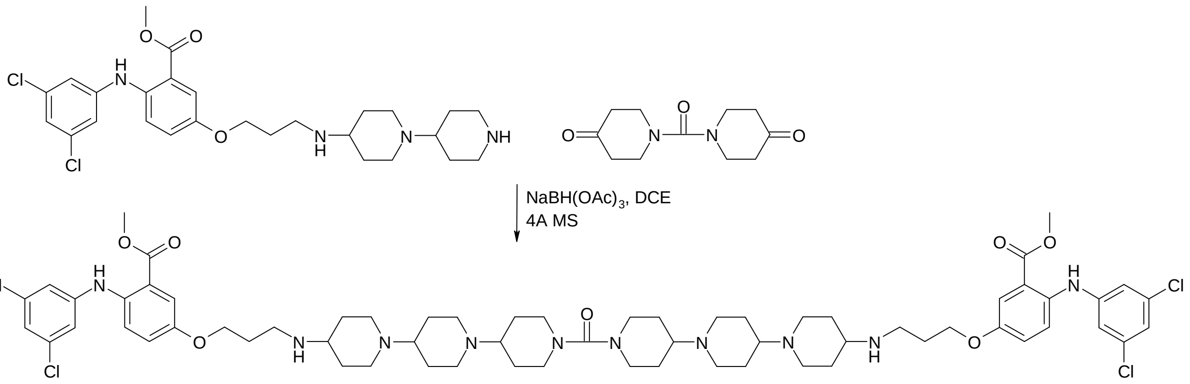

5-[3-([1,4']Bipiperidinyl-4-ylamino)-propoxy]-2-(3,5-dichloro-phenylamino)-benzoic acid methyl ester (263 mg, 0.491 mmol) was combined with bis-(4-oxapiperidyl)-methanone (55 mg, 0.245 mmol) in dichloroethane (4 mL), and 4 Å molecular sieves (300 mg) and sodium triacetoxyborohydride (312 mg, 1.472 mmol) were added. The reaction was allowed to stir for 4 days and then quenched with sodium hydrogencarbonate and extracted three times with dichloromethane. The combined organics were washed with brine and then dried over sodium sulfate. The residue was then purified by reverse phase HPLC using a gradient of 80%

(0.05% formic acid in water)/methanol to 20% (0.05% formic acid in water)/methanol. The pure fractions were combined and concentrated *in vacuo* to afford the title compound (62 mg), a yellow solid, as the formate salt. ESMS $[M+H]^{+}$ = 1261.60. ^{1}H NMR (300 MHz, MeOH-D_4) δ: 7.58 (2H, d, *J* = 3.0 Hz), 7.34 (2H, d, *J* = 8.8 Hz), 7.17 (2H, dd, *J* = 8.9, 2.7 Hz), 7.01 (4H, d, *J* = 1.8 Hz), 6.94-6.90 (2H, m), 4.18-4.07 (4H, m), 3.93-3.73 (10H, m), 3.61-3.48 (4H, m), 3.35-3.12 (16H, m), 3.06-2.73 (10H, m), 2.54-2.39 (4H, m), 2.27-2.03 (16H, m), 2.01-1.84 (4H, m), 1.82-1.62 (8H, m).

<u>IIa</u>

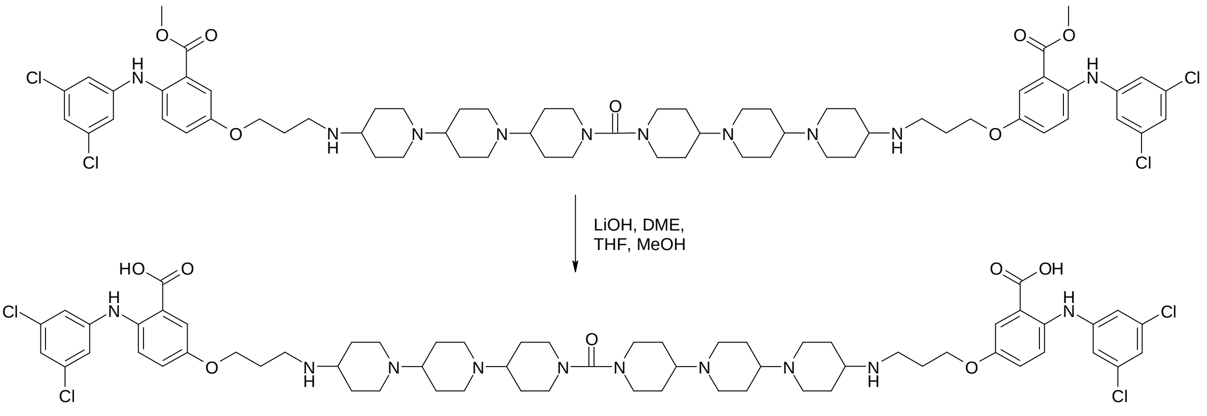

IIa methyl ester (formate salt, 62 mg, 0.043 mmol) was dissolved in dimethoxyethane (1 mL), tetrahydrofuran (1 mL) and methanol (1 mL) and lithium hydroxide added (0.5 M in water, 191 µL, 0.095 mmol) was added. The mixture was heated to 70°C for 3 h. LCMS showed no change, so additional LiOH (0.5 M in water, 191 µL, 0.095 mmol) was added and the mixture allowed to stir at 70°C overnight. LCMS showed no change so additional LiOH (0.5 M in water, 1 mL) was added. After 3 h the reaction showed complete conversion to the di-acid. The mixture was allowed to cool to room temperature. The mixture was quenched with 2N HCl and a yellow precipitate appeared. The mixture was diluted with MeOH and loaded onto a SCX cartridge. The cartridge was washed with methanol, then the product was eluted with 0.880 NH_3 in MeOH (10%). The fraction containing product was concentrated *in vacuo* and dried under high vacuum at 70°C to give the title compound (12.1 mg) as a pale yellow powder. ESMS $[M+H]^{++}/2$ = 618.69. ^{1}H NMR (300 MHz, MeOH-D_4/DMSO-D6) δ: 7.50 (2H, d, *J* = 3.3 Hz), 7.25 (2H, d, *J* = 9.0 Hz), 7.01 (2H, dd, *J* = 9.0, 2.2 Hz), 6.95 (4H, d, *J* = 1.7 Hz), 6.84-6.81 (2H, m), 4.09-4.02 (10H, m), 3.72-3.61 (4H, m), 3.52-3.39 (4H, m), 3.36-3.27 (4H, m) 3.2-2.64 (10H, m), 2.31-2.19 (4H, m), 2.13-1.44 (30H, m).

Bis-[4-(1,4-dioxa-8-aza-spiro[4.5]dec-8-yl)-piperidin-1-yl]-methanone

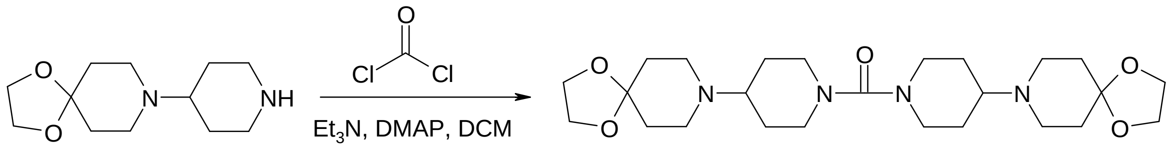

1,4-Dioxa-8-azaspiro[4.5]decane-8-(4-piperidinyl) (548 mg, 2.42 mmol) was dissolved in dichloromethane and triethylamine (0.67 mL, 4.84 mmol) was added. Under N_2, phosgene (0.75 mL, 1.45 mmol) was added, followed by 4-dimethylaminopyridine (5 mg). After 2 h, the reaction was quenched with water, then extracted 3 x with dichloromethane. The combined organics were washed with brine, dried over sodium sulfate, filtered and concentrated *in vacuo*. The solid was triturated with diethyl ether and hexanes, but this did not totally remove the impurities. Purification by flash column chromatography with 5% (10% 0.880 NH_3 in methanol)/dichloromethane gave the title compound (338 mg) as a colourless solid. ESMS $[M+H]^+$ = 479.33. ^1H NMR (300 MHz, $CDCl_3$) δ: 3.90 (8H, s), 3.69 (4H, d, *J* = 13.3 Hz), 2.68 (4H, dd, *J* = 12.2, 12.2 Hz), 2.61-2.56 (8H, m), 2.47-2.35 (2H, m), 1.81-1.64 (12H, m), 1.52-1.35 (4H, m).

Diketone

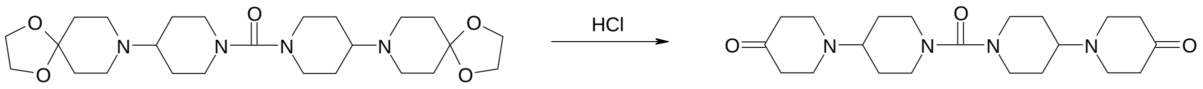

Bis-[4-(1,4-dioxa-8-aza-spiro[4.5]dec-8-yl)-piperidin-1-yl]-methanone (338 mg, 0.706 mmol) was dissolved in 5N HCl and stirred for 16 h. The reaction was cooled to 0°C and quenched by the slow addition of 2N NaOH. The mixture was then extracted with ethyl acetate x 3. The combined organics were washed with brine, dried over sodium sulfate and concentrated *in vacuo* to give colourless crystals. Purification by flash column chromatography using 10% (10% 0.880 NH_3 in methanol)/dichloromethane gave the title compound (210 mg) as a colourless foam. ESMS $[M+H]^+$ = 391.27. ^1H NMR (300 MHz, $CDCl_3$) 3.75-3.64 (4H, m), 2.86-2.50 (16H, m), 2.43-2.34 (6H, m), 1.82-1.72 (4H, m), 1.55-1.38 (4H, m).

IId Dimethyl ester

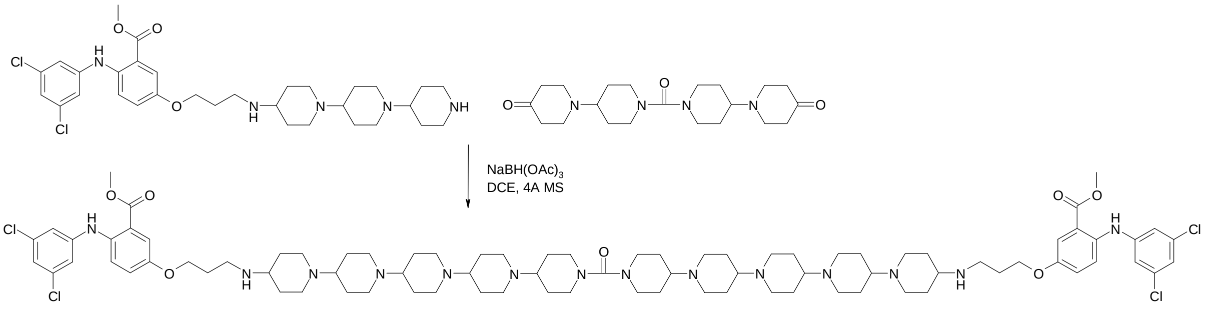

Method as for IIc dimethyl ester using 2-(3,5-dichloro-phenylamino)-5-[3-([1,4';1',4"]terpiperidin-4-ylamino)-propoxy]-benzoic acid methyl ester (269 mg, 0.435 mmol) and the diketone (85 mg, 0.217 mmol) instead of bis-(4-oxapiperidyl)-methanone. The title compound (41 mg) was obtained as a pale yellow foam. ESMS $[M+H]^{++}/2$ = 797.75. ^1H NMR (300 MHz, $CDCl_3$/MeOH-D4) δ: 7.53-7.44 (2H, m), 7.34-7.27 (2H, m), 7.11-7.04 (2H, m), 7.03-6.97 (4H, m) 6.93-6.87 (2H, m), 4.12-3.69 (14H, m), 3.49-3.05 (28H, m), 3.01-2.41 (24H, m), 2.26-1.48 (44H, m).

IId

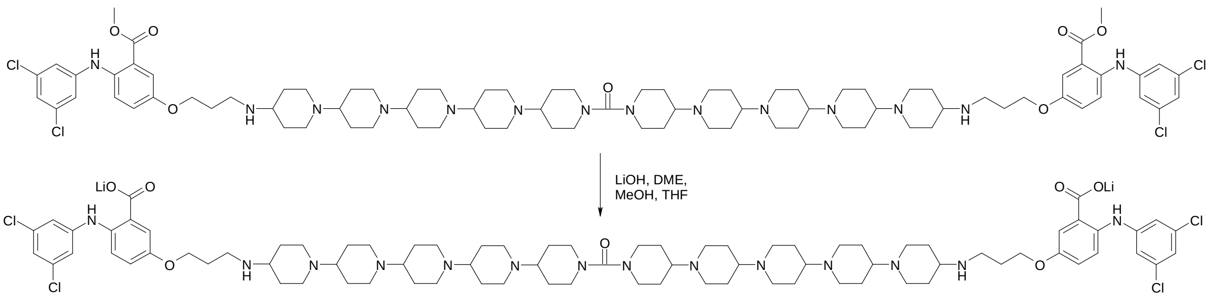

IId dimethyl ester (41 mg, 0.0267 mmol) was dissolved in methanol (1 mL), dimethoxyethane (1 mL) and tetrahydrofuran (1 mL). LiOH (0.5 M in water, 0.64 mL, 0.32 mmol) was added and the mixture was heated at 70°C for 16 h. A precipitate had formed. The mixture was cooled to room temperature and filtered. The solid was dried under high vacuum to afford the title compound (38 mg) as a colourless solid as the di lithium salt. ESMS $[M+H]^{++}/2$ = 783.72. ^1H NMR (300 MHz, MeOH-D4) δ: 7.56 (2H, d, J = 3.0 Hz), 7.27 (2H, d, J = 8.8 Hz), 7.01-6.93 (6H, m), 6.80-6.77 (2H, m), 4.12-4.05 (4H, m), 3.80-3.68 (4H, m), 3.12-2.74 (24H, m), 2.63-2.40 (4H, m), 2.35-2.12 (28H, m), 2.05-1.81 (26H, m), 1.64-1.37 (20H, m).

2-(3,5-Dichloro-phenylamino)-5-(piperidin-4-ylmethoxy)-benzoic acid methyl ester hydrochloride

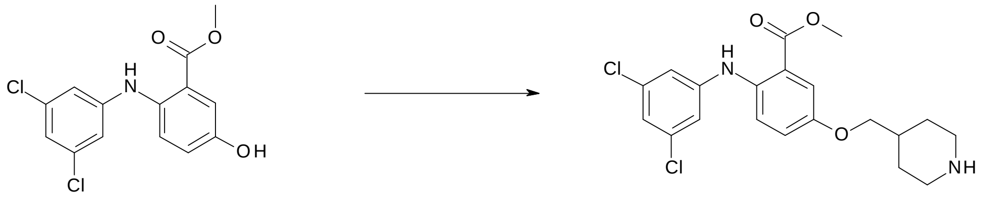

To a stirred solution of 2-(3,5-dichloro-phenylamino)-5-hydroxy-benzoic acid methyl ester (0.9 g, 2.88 mmol), boc-piperidinol (0.7 g, 3.5 mmol) triphenyl phosphine (0.91 g, 3.5 mmol) in anhydrous THF (10 mL) at 0°C, was added DIAD (0.68 mL, 3.5 mmol) dropwise. The resulting mixture was left to stir at 0°C for 2 h and then at room temperature for 17 h. It was quenched with the addition of ice/water and extracted with ethyl acetate. The combined organic layer was washed with brine, dried with sodium sulfate, filtered and the filtrate was evaporated under reduced pressure to give red oil. Silica gel chromatography eluting with ethyl acetate in *i*-hexanes (5-10%) gave the desired boc-piperidine (0.16 g) as amber oil. A solution of the amber oil in 4M HCl in dioxane (6 mL) was left to stir for 6 h. It was evaporated under reduced pressure. The crude solid was triturated in diethyl ether/hexane (1:1). The resulting solid was collected by filtration and dried to give the title compound as colourless solid (0.165 g). ESMS $[M+H]^+$ = 409.1. ^{1}H NMR (300 MHz, DMSO-D6) δ: 8.80 (1H, br.s), 8.67 (1H, s), 7.45-7.30 (2H, m), 7.25-7.15 (1H, m), 6.97-6.87 (3H, m), 3.95-3.83 (2H, m), 3.80 (3H, s), 3.00-2.82 (2H, m), 2.04 (1H, m), 1.98-1.85 (2H, m), 1.60-1.40 (2H, m).

4-[4-(3,5-Dichloro-phenylamino)-3-methoxycarbonyl-phenoxymethyl]-[1,4';1',4'';1'',4''']quaterpiperidine-1'''-carboxylic acid tert-butyl ester

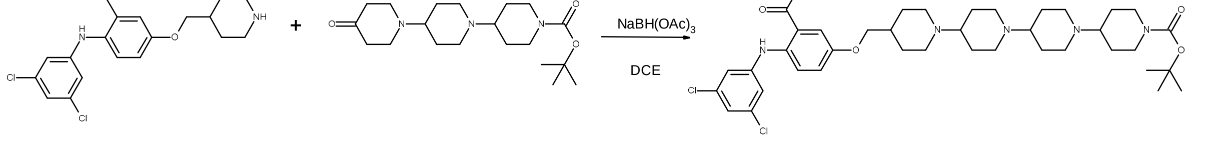

A mixture of 2-(3,5-dichloro-phenylamino)-5-(piperidin-4-ylmethoxy)-benzoic acid methyl ester (0.88 g, 2 mmol), 4-oxo-[1,4';1',4'']terpiperidine-1''-carboxylic acid tert-butyl ester (0.88

g, 2.4 mmol) and sodium triacetoxyborohydride (0.85 g, 4 mmol) in anhydrous 1,2-dichloroethane (6 mL) was left to stir for 4 days at room temperature. It was evaporated under reduced pressure. The crude residue was stirred in sodium hydroxide (1 M, 30 mL) for 30 min. The mixture was extracted with DCM. The combined organic layer was washed with brine, dried with sodium sulfate, filtered and the filtrate was evaporated under reduced pressure to give solid foam which was triturated in ethyl acetate (30 mL). The undissolved solid was collected by filtration and air-dried to give the title compound as cream solid (0.81 g, 53%). ESMS $[M+H]^+$ = 758.57. ^{1}H NMR (300 MHz, MeOH-D4) δ: 7.52 (1H, d, *J* = 2 Hz), 7.35 (1H, d, *J* = 8 Hz), 7.15 (1H, dd, *J* = 8, 2 Hz), 7.03 (2H, s), 6.90 (1H, s), 4.21-4.05 (2H, m), 3.91 (3H, s), 3.89-3.82 (2H, m), 3.15-3.00 (6H, m), 2.85-2.65 (2H, m), 2.55-2.40 (1H, m), 2.40-2.15 (8H, m), 1.98-1.75 (9H, m), 1.25-1.70 (17H, m).

2-(3,5-Dichloro-phenylamino)-5-([1,4';1',4'';1'',4''']quaterpiperidin-4-ylmethoxy)-benzoic acid methyl ester

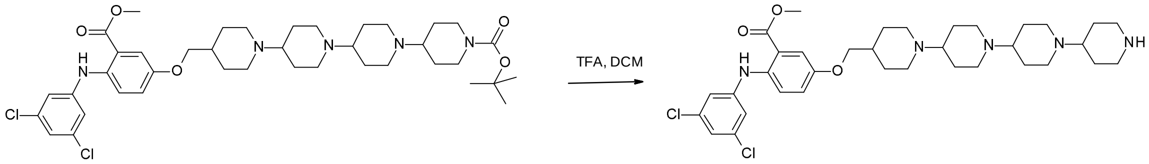

Trifluoroacetic acid (10 mL) was added to a solution of 4-[4-(3,5-dichloro-phenylamino)-3-methoxycarbonyl-phenoxymethyl]-[1,4';1',4";1",4"']quaterpiperidine-1"'-carboxylic acid tert-butyl ester (0.81 g, 1.07 mmol) in DCM (20 mL). The resulting solution was left to stir for 4 h before it was evaporated to give dry foam. The latter was partitioned between sodium hydroxide (1 M, 40 mL) and 5% MeOH in DCM. The aqueous layer was separated and extracted with 5% MeOH in DCM. The combined organic layer was washed with brine, dried with magnesium sulfate, filtered and the filtrate was evaporated under reduced pressure to give yellow solid. It was applied onto a pre-conditioned SCX cartridge. The cartridge was washed with DCM and then methanol followed by ammonia in methanol (1M)/DCM (9:1). Evaporation of the ammoniacal fraction yielded the title product as yellow solid (0.15 g, 21%). ESMS $[M+H]^+$ = 658.50.^{1}H NMR (300 MHz, $CDCl_3$) δ: 9.02 (1H, s), 7.46 (1H, d, *J* = 2 Hz), 7.30 (1H, d, *J* = 8 Hz), 7.05 (1H, dd, *J* = 8, 2 Hz), 7.01 (2H, s), 6.90 (1H, s), 3.92 (3H, s), 3.87-3.78 (2H, m), 3.20-3.10 (2H, m), 3.05-2.90 (6H, m), 2.70-2.50 (2H, m), 2.45-2.10 (8H, m), 1.95-1.30 (19H, m).

4-[4-(3,5-Dichloro-phenylamino)-3-methoxycarbonyl-phenoxymethyl]-[1,4';1',4'';1'',4''';1''',4'''']quinquepiperidine-1''''-carboxylic acid 4-nitro-phenyl ester

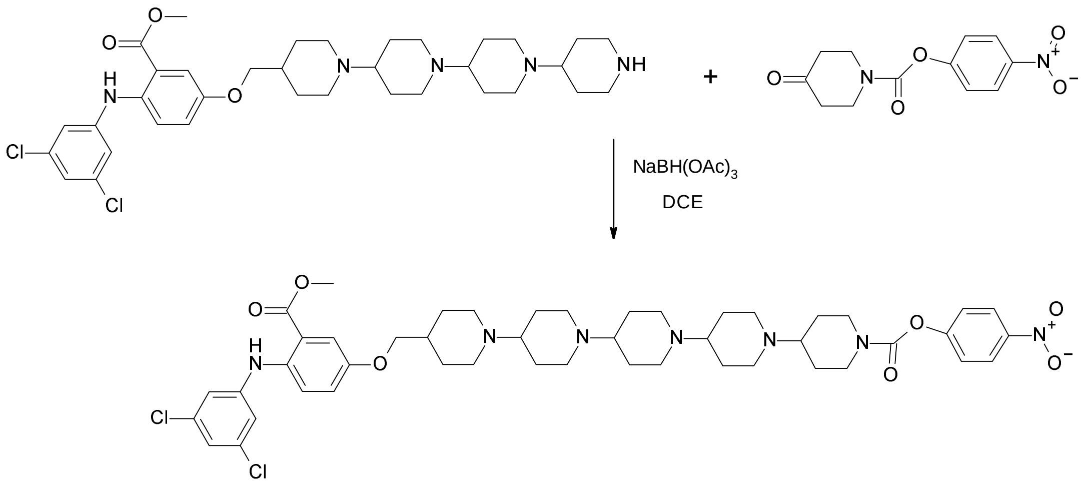

2-(3,5-Dichloro-phenylamino)-5-([1,4';1',4";1",4'"]quaterpiperidin-4-ylmethoxy)-benzoic acid methyl ester (0.355 g, 0.54 mmol), 4-oxo-piperidine-1-carboxylic acid 4-nitro-phenyl ester (0.17 g, 0.65 mmol) and sodium triacetoxyborohydride (0.22 g, 0.75 mmol) in anhydrous 1,2-dichloroethane (10 mL) was left to stir for 3 days at room temperature. It was evaporated under reduced pressure. The crude residue was partitioned between 1 M sodium hydroxide and DCM. The organic layer was collected by means of a phase separation cartridge and evaporated to give yellow residue. The latter was applied to a pre-washed SCX cartridge. The cartridge was washed with DCM and then methanol followed by ammonia in methanol (1M)/DCM (9:1). Evaporation of the ammoniacal fraction yielded titled product as yellow solid (0.42 g, 86%). ESMS $[M+H]^+$ = 906.66. ^{1}H NMR (300MHz, $CDCl_3$) δ: 9.02 (1H, s), 8.25 (1H, d, *J* = 8 Hz), 7.47 (1H, d, *J* = 2 Hz), 7.35-7.25 (3H, m), 7.05 (1H, dd, *J* = 8, 2 Hz), 7.01 (2H, s), 6.90 (1H, s), 4.35-4.20 (2H, m), 3.92 (3H, s), 3.87-3.78 (2H, m), 3.05-2.90 (10H, m), 2.50 (1H, m), 2.45-2.10 (9H, m), 2.0-1.30 (32H, m).

4-[4-(3,5-Dichloro-phenylamino)-3-methoxycarbonyl-phenoxymethyl]-[1,4']bipiperidinyl-1'-carboxylic acid tert-butyl ester

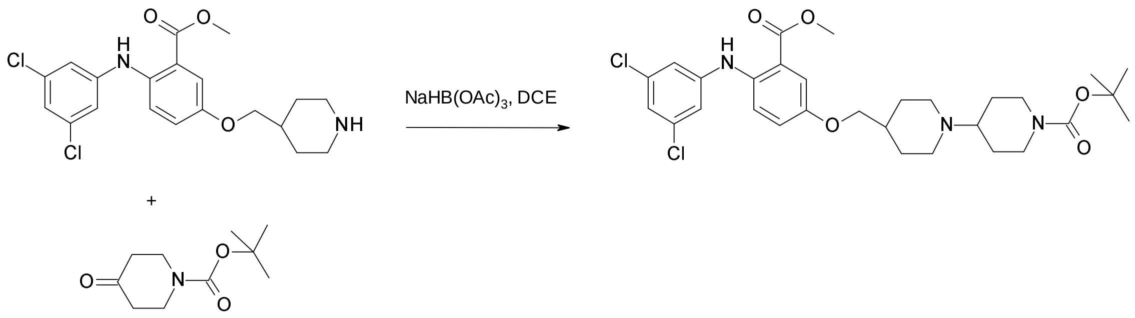

A mixture of 2-(3,5-dichloro-phenylamino)-5-(piperidin-4-ylmethoxy)-benzoic acid methyl ester (0.47 g, 1.15 mmol), 1-boc-4-piperidone (0.297 g, 1.49 mmol) and sodium triacetoxyborohydride (0.54 g, 1.84 mmol) in anhydrous 1,2-dichloroethane (10 mL) was left to stir for 2 days at room temperature. It was evaporated under reduced pressure. The crude residue was partitioned between sodium hydroxide and DCM. The aqueous layer was extracted with DCM. The combined organic layer was washed with brine, dried with sodium sulfate, filtered and the filtrate was evaporated under reduced pressure to give crude oil. Silica gel chromatography eluting with methanol (containing 10% 0.880 NH_3) in ethyl acetate (0-5%) give amber oil. It was then applied onto a pre-conditioned SCX cartridge. The cartridge was washed with DCM and then methanol followed by ammonia in methanol (1M)/DCM (9:1). Evaporation of the ammoniacal fraction yielded the title product as amber oil (0.49 g, 56%). ESMS $[M+H]^+$= 592.24. ^{1}H NMR (300 MHz, $CDCl_3$) δ: 9.05 (1H, br.s), 7.47 (1H, d, *J* = 2 Hz), 7.31 (1H, d, *J* = 8Hz), 7.05 (1H, dd, *J* = 8, 2 Hz), 7.03 (2H, s), 6.90 (1H, s), 4.27-4.10 (1H, m), 3.91 (3H, s), 3.92 - 3.80 (2H, m), 3.22-3.08 (1H, m), 3.05-2.90 (2H, m), 2.80-2.55 (1H, m), 2.53-2.30 (1H, m), 2.32-2.15 (2H, m), 1.96-1.70 (8H, m), 1.60-1.30 (11H, m).

5-([1,4']Bipiperidinyl-4-ylmethoxy)-2-(3,5-dichloro-phenylamino)-benzoic acid methyl ester

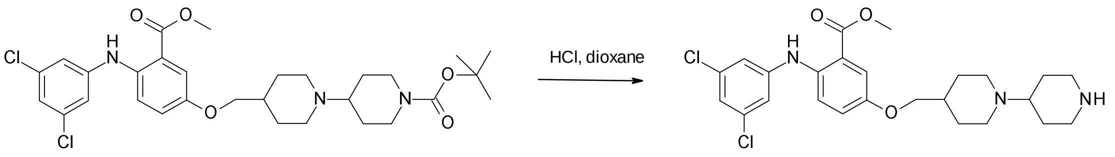

4 M HCl in dioxane (6 mL) was added to a solution of 4-[4-(3,5-dichloro-phenylamino)-3-methoxycarbonyl-phenoxymethyl]-[1,4']bipiperidinyl-1'-carboxylic acid tert-butyl ester (0.49 g, 0.83 mmol) in dioxane (2 mL). The resulting mixture was left to stir for 17 h at room temperature before it was evaporated to give dry foam. It was applied onto a pre-conditioned SCX cartridge. The cartridge was washed with DCM and then methanol followed by ammonia in methanol (0.5 M). Evaporation of the ammoniacal fraction yielded title product as yellow solid (0.42 g, 100%). ESMS $[M+H]^+ = 492.4$. ^1H NMR (300 MHz, $CDCl_3$) δ: 9.05 (1H, br.s), 7.50 (1H, d, *J* = 2 Hz), 7.30 (1H, d, *J* = 8Hz), 7.13-7.01 (3H, m), 6.90 (1H, s), 3.92 (3H, s), 3.90-3.75 (2H, m), 3.25 -3.12 (2H, m), 3.10-2.95 (2H, m), 2.75-2.60 (2H, m), 2.55-2.37 (1H, m), 2.35-2.20 (2H, m), 1.96 -1.60 (6H, m), 1.60-1.30 (4H, m).

<u>IIe</u>

5-(1''''-{4-[4-(3,5-Dichloro-phenylamino)-3-carboxy-phenoxymethyl]-[1,4']bipiperidinyl-1'-carbonyl}-[1,4';1',4'';1'',4''';1''',4'''']quinquepiperidin-4-ylmethoxy)-2-(3,5-dichloro-phenylamino)-benzoic acid di-lithium salt

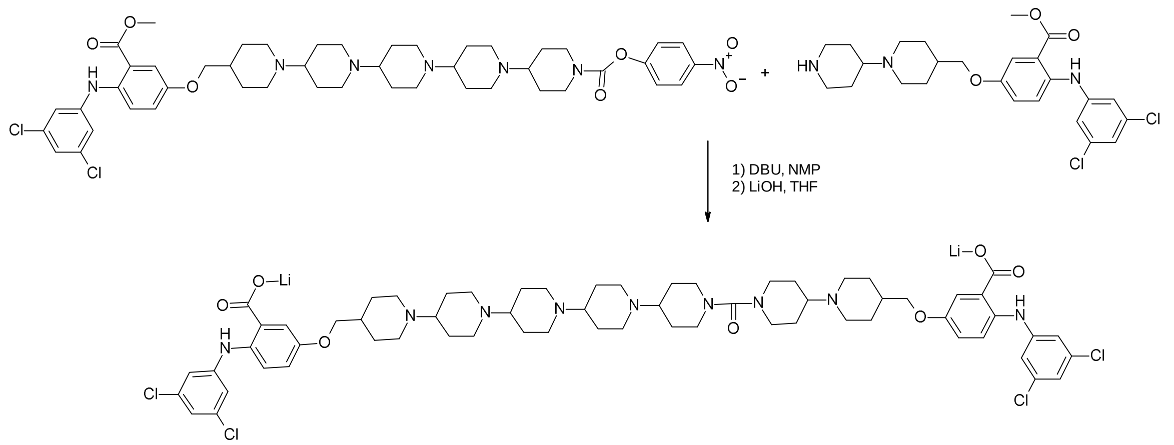

A stream of anhydrous nitrogen was bubbled into a solution of 2-(3,5-dichloro-phenylamino)-5-[1''''-(4-nitro-benzoyl)-[1,4';1',4'';1'',4''';1''',4'''']quinquepiperidin-4-ylmethoxy]-benzoic acid methyl ester (0.23 g, 0.25 mmol), 5-([1,4']bipiperidinyl-4-ylmethoxy)-2-(3,5-dichloro-phenylamino)-benzoic acid methyl ester (0.13 g, 0.25 mmol) and DBU (0.11 mL, 0.75 mmol) in anhydrous NMP (2 mL) in a sealed microwave reaction vessel for 15 min. The mixture was heated in a microwave reactor at 140°C for 2.3 h. The reaction mixture was allowed to cool to room temperature and purified by semi-preparative HPLC (Sample solution: crude reaction mixture in NMP, Injection volume: 80uL, Column: Kromasil C18 100x10mm, Mobile phase A: 1% formic acid in water, B: 1% formic acid in methanol, Method: 10% B to 100% B over 15 mins and at 100% B for 2 mins, Flow rate: 8 mL/min, Detection: UV at 295 nm). Product fractions obtained were a mixture of the ester and carboxylic acids and were evaporated under reduced pressure to give colourless solid. The latter was left to stir in 0.5 M LiOH (0.5 mL) and THF (0.3 mL) for 17 h. The mixture was diluted with water (0.5 mL) and then centrifuged. The supernatant was removed and the remaining solid was dried in a vacuum oven to give the titled compound as pale yellow solid (25 mg, 8%). ESMS $[M+H]^+$ = 1230.7. ^{1}H NMR (300 MHz, MeOH-D4) δ: 7.60-7.50 (2H, d, *J* = 2 Hz), 7.30-7.20 (2H, d, *J* = 8 Hz), 7.05 -6.90 (6H, m), 6.75 (2H, s), 3.95-3.70 (8H, m), 3.12-2.95 (8H, m), 2.90-2.70 (4H, m), 2.60-2.40 (2H, m), 2.40-2.10 (9H, m), 2.00-1.35 (36H, m).

4-(1,4-Dioxa-8-aza-spiro[4.5]dec-8-yl)-[1,4';1',4'']terpiperidine-1''-carboxylic acid benzyl ester

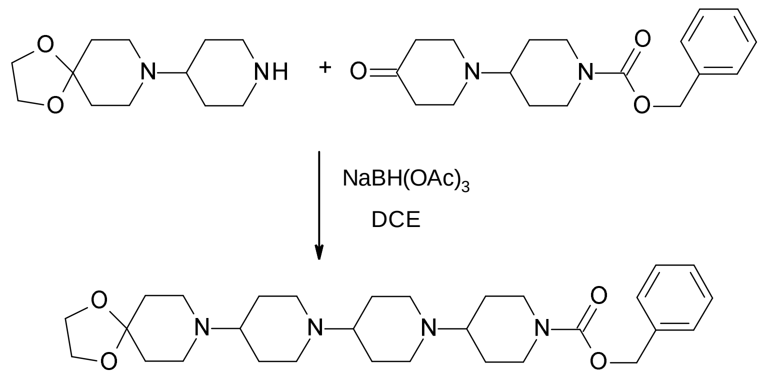

A mixture of 4-oxo-[1,4']bipiperidinyl-1'-carboxylic acid benzyl ester (0.56 g, 1.77 mmol), 8-piperidin-4-yl-1,4-dioxa-8-aza-spiro[4.5]decane (0.4 g, 1.77 mmol) and sodium

triacetoxyborohydride (0.45 g, 2.12 mmol) in anhydrous 1,2-dichloroethane (7 mL) and glacial acetic acid (0.21 g, 3.54 mmol) was left to stir for 17 h at room temperature. It was evaporated under reduced pressure. The crude oil was partitioned between saturated solution of sodium hydrogen carbonate and DCM. The aqueous layer was extracted with DCM. The combined organic layer was washed with brine, dried with sodium sulfate, filtered and the filtrate was evaporated under reduced pressure to give a crude oil. Trituration of the latter in diethyl ether gave a colourless solid precipitate which was collected by filtration and dried under reduced pressure to give the titled product (0.56 g, 60%). ^{1}H NMR. (300 MHz, $CDCl_3$) δ: 7.40-7.30 (5H, m), 5.11 (2H, s), 4.35-4.15 (2H, m), 3.94 (4H, s), 3.10-2.92 (4H, m), 2.90-2.72 (2H, m), 2.70-2.62 (4H, m), 2.50-2.10 (7H, m), 1.90-1.40 (16H, m).

4-Oxo-[1,4';1',4'';1'',4''']quaterpiperidine-1'''-carboxylic acid benzyl ester

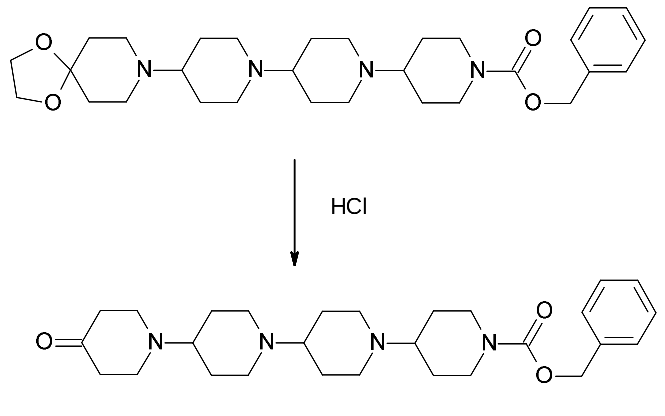

A solution of 4-(1,4-dioxa-8-aza-spiro[4.5]dec-8-yl)-[1,4';1',4'']terpiperidine-1''-carboxylic acid benzyl ester (1.73 g, 3.28 mmol) in hydrochloric acid (5 M)/THF (60 mL, 1:1) was heated under reflux for 3 h. It was cooled in an ice-bath and basified to pH 10 with the addition of sodium hydroxide (4 M). The contents were extracted with ethyl acetate. The combined organic layer was washed with brine, dried with sodium sulfate, filtered and the filtrate was evaporated under reduced pressure to give a crude oil. It was triturated in diethyl ether to give a colourless solid precipitate which was collected by filtration and dried under reduced pressure to give the titled product (0.99 g, 62%). ESMS $[M+H]^+$ = 483.4. ^{1}H NMR (300 MHz, $CDCl_3$) δ: 7.40-7.30 (5H, m), 5.11 (2H, s), 4.30-4.10 (2H, m), 3.05-2.70 (10H, m), 2.50-2.10 (9H, m), 1.90-1.30 (14H, m).

4-Oxo-[1,4';1',4'';1'',4''']quaterpiperidine-1'''-carboxylic acid tert-butyl ester

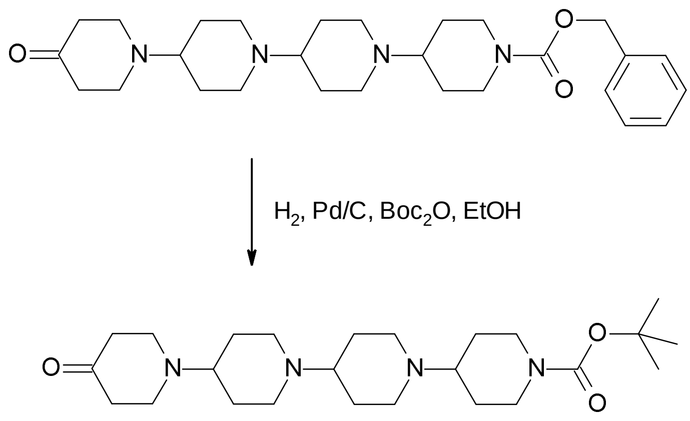

10% Palladium on charcoal (0.1 g) was added to a solution of 4-oxo-[1,4';1',4";1",4'"] quaterpiperidine-1'"-carboxylic acid benzyl ester (0.77 g, 1.6 mmol) and boc-anhydride (0.45 g, 2 mmol) in ethanol (40 mL). The resulting mixture was left to stir under atmospheric hydrogen for 18 h. The catalyst was removed on a bed of hyflo and the filtrate was evaporated to give crude oil. It was applied onto a pre-conditioned SCX cartridge. The cartridge was washed with DCM and then methanol followed by ammonia in methanol (1 M). Evaporation of the ammoniacal fraction yielded the title product as amber oil (0.51 g, 71%). ESMS $[M+H]^+$ = 449.3. ^1H NMR (300 MHz, $CDCl_3$) δ: 4.25-4.05 (2H, m), 3.10-2.85 (8H, m), 2.80-2.60 (2H, m), 2.55-2.10 (11H, m), 1.90-1.30 (21H, m).

4-{3-[4-(3,5-Dichloro-phenylamino)-3-methoxycarbonyl-phenoxy]-propylamino}-[1,4';1',4'';1'',4''']quaterpiperidine-1'''-carboxylic acid tert-butyl ester

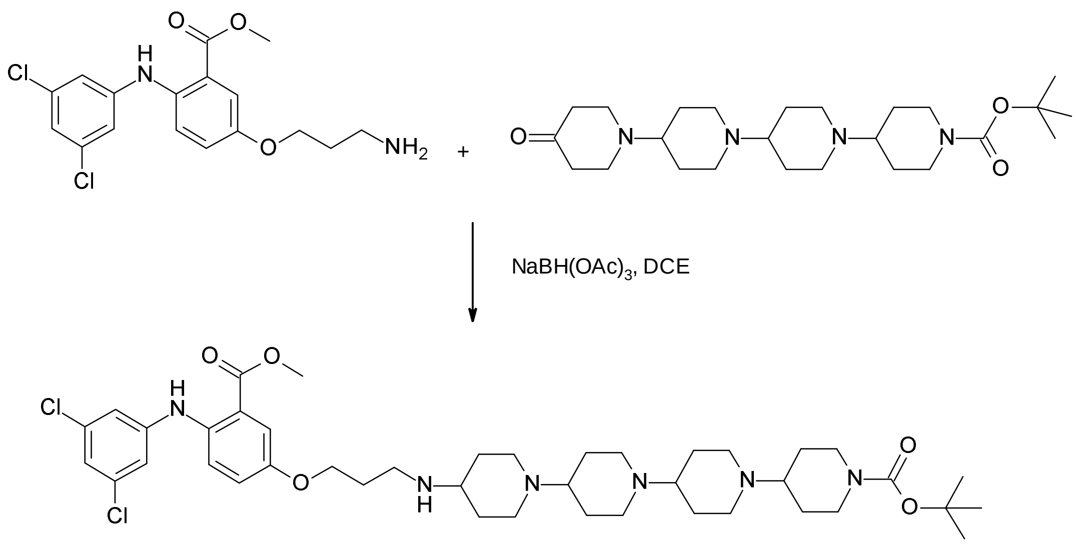

A mixture of 5-(3-amino-propoxy)-2-(3,5-dichloro-phenylamino)-benzoic acid methyl ester (0.14 g, 0.38 mmol), 4-oxo-[1,4';1',4";1",4"']quaterpiperidine-1"'-carboxylic acid tert-butyl ester (0.17 g, 0.38 mmol) and sodium triacetoxyborohydride (0.14 g, 0.49 mmol) in anhydrous 1,2-dichloroethane (6 mL) was left to stir for 24 h at room temperature. It was evaporated under reduced pressure. The crude residue was partitioned between sodium hydroxide (0.5 M) and DCM. The aqueous layer was extracted with DCM. The combined organic layer was washed with brine, dried with sodium sulfate, filtered and the filtrate was evaporated under reduced pressure to give the titled compound as colourless solid (0.32 g, 100%). ESMS $[M+H]^+$ = 801.6. ^1H NMR (300 MHz, $CDCl_3$) δ: 9.10 (1H, s), 7.48 (1H, d, *J* = 2 Hz), 7.32 (1H, d, *J* = 8 Hz), 7.00-7.15 (3H, m), 6.92 (1H, s), 4.15 (2H, m), 4.06 (2H, m), 3.90 (3H, s), 1.25-3.00 (48H, m).

2-(3,5-Dichloro-phenylamino)-5-[3-([1,4';1',4'';1'',4''']quaterpiperidin-4-ylamino)-propoxy]-benzoic acid methyl ester

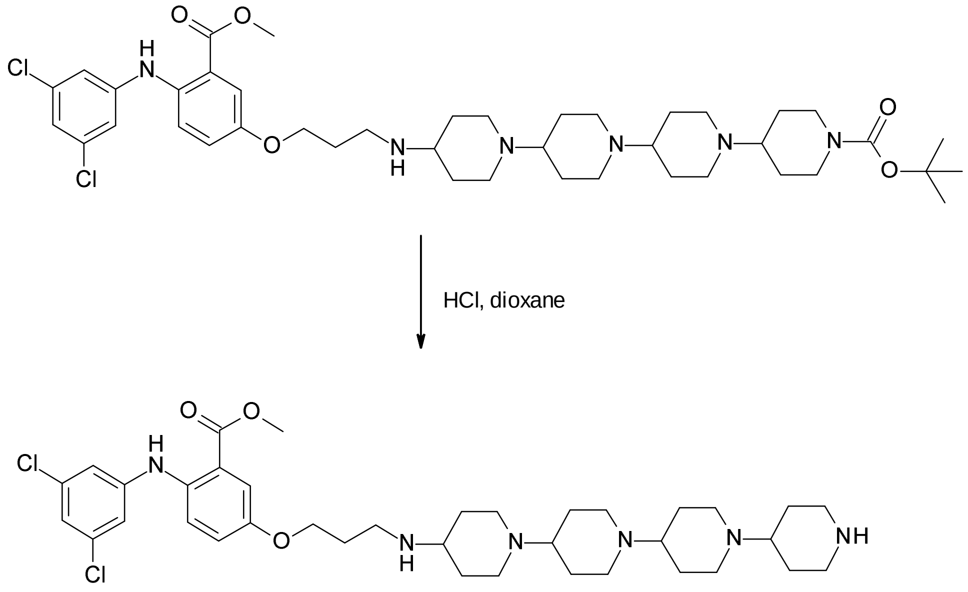

4 M HCl in dioxane (6 mL) was added to a solution of 4-{3-[4-(3,5-dichloro-phenylamino)-3-methoxycarbonyl-phenoxy]-propylamino}-[1,4';1',4";1",4"']quaterpiperidine-1"'-carboxylic acid tert-butyl ester
(0.32 g, 0.39 mmol) in dioxane (2 mL). The resulting mixture was left to stir for 17 h at room temperature before it was evaporated to give dry foam. It was treated with sodium hydroxide (1 M, 10 mL) and methanol (20 mL) for 20 mins. The mixture was evaporated down and treated with water (50 mL). The solid was collected by filtration, air-dried and then triturated

in methanol (10 mL). The purified solid was again filtered and dried under reduced pressure to yield the titled product as yellow solid (0.105 g, 38%). ESMS $[M+H]^+$ = 701.5. ^{1}H NMR (300 MHz, DMSO-D6) δ: 8.62 (1H, s), 7.25-7.40 (2H, m), 7.15 (1H, m), 6.85-7.00 (3H, m), 4.05 (2H, m), 3.80 (3H, s), 1.25-3.00 (42H, m).

4-Oxo-piperidine-1-carboxylic acid 4-nitro-phenyl ester

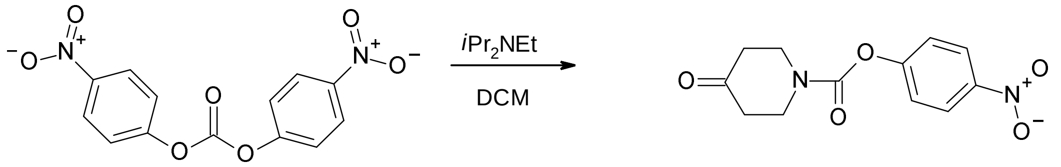

Diisopropylethylamine (0.52 g, 4 mmol) was added to a solution of carbonic acid bis-(4-nitro-phenyl) ester (0.61 g, 2 mmol) and 4-piperidone hydrochloride monohydrate (0.31 g, 2 mmol) in DCM (10 mL). Resulting solution was left to stir for 24 h before it was evaporated to give yellow oil. Silica gel chromatography eluting with DCM gave impure solid which was partitioned between NaOH (0.5 M) and DCM. The organic layer was collected through a phase-separation cartridge and evaporated under reduced pressure to give the title compound as colourless solid (0.35 g, 65%). ^{1}H NMR (300 MHz, $CDCl_3$) δ: 8.25 (2H, d, *J* = 8 Hz), 7.33 (2H, d, *J* = 8Hz), 3.85-4.10 (4H, m), 2.25-2.70 (4H, m).

4-{3-[4-(3,5-Dichloro-phenylamino)-3-methoxycarbonyl-phenoxy]-propylamino}-piperidine-1-carboxylic acid 4-nitro-phenyl ester

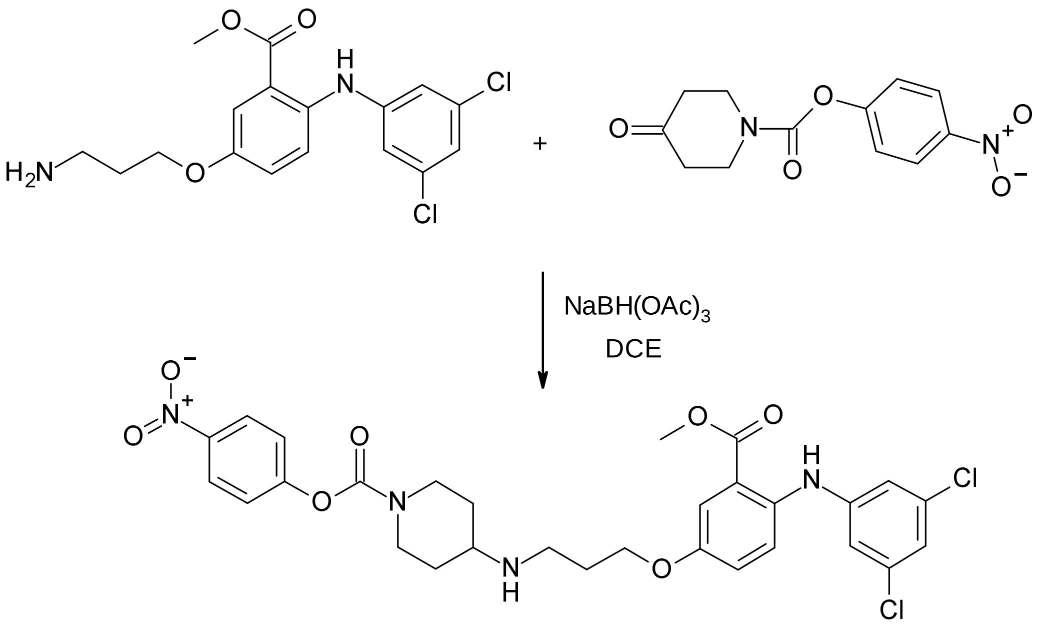

A mixture of 5-(3-amino-propoxy)-2-(3,5-dichloro-phenylamino)-benzoic acid methyl ester (0.41 g, 1.1 mmol), 4-oxo-piperidine-1-carboxylic acid 4-nitro-phenyl ester (0.35 g, 1.32 mmol) and sodium triacetoxyborohydride (0.45 g, 1.5 mmol) in anhydrous 1,2-dichloroethane (8 mL) was left to stir for 24 h at room temperature. It was evaporated under reduced pressure. The crude residue was partitioned between sodium hydroxide (0.5 M) and DCM. The aqueous layer was extracted with DCM. The combined organic layer was washed with brine, dried with sodium sulfate, filtered and the filtrate was evaporated under reduced pressure to give crude oil. Silica gel chromatography eluting with ethyl acetate in hexane (0-100%) give the title compound as amber oil (0.56 g, 68%). ESMS $[M+H]^+$ = 617.35. ^{1}H NMR (300 MHz, $CDCl_3$) δ: 9.08 (1H, s), 8.23 (2H, d, *J* = 8 Hz), 7.50 (1H, d, *J* = 2 Hz), 7.25-7.40 (3H, m), 7.00-7.15 (3H, m), 6.93 (1H, s), 4.02-4.30 (4H, m), 3.91 (3H, s), 2.98-3.25 (2H, m), 2.90 (1H, m), 2.78 (1H, m), 1.93-2.10 (4H, m),1.30-1.50 (3H, m).

IIb

5-{3-[1'''-(4-{3-[4-(3,5-Dichlorophenylamino)-3-Carboxy-phenoxy]-propylamino}-piperidine-1-carbonyl)-[1,4';1',4'';1'',4''']quaterpiperidin-4-ylamino]-propoxy}-2-(3,5-dichloro-phenylamino)-benzoic acid dilithium salt

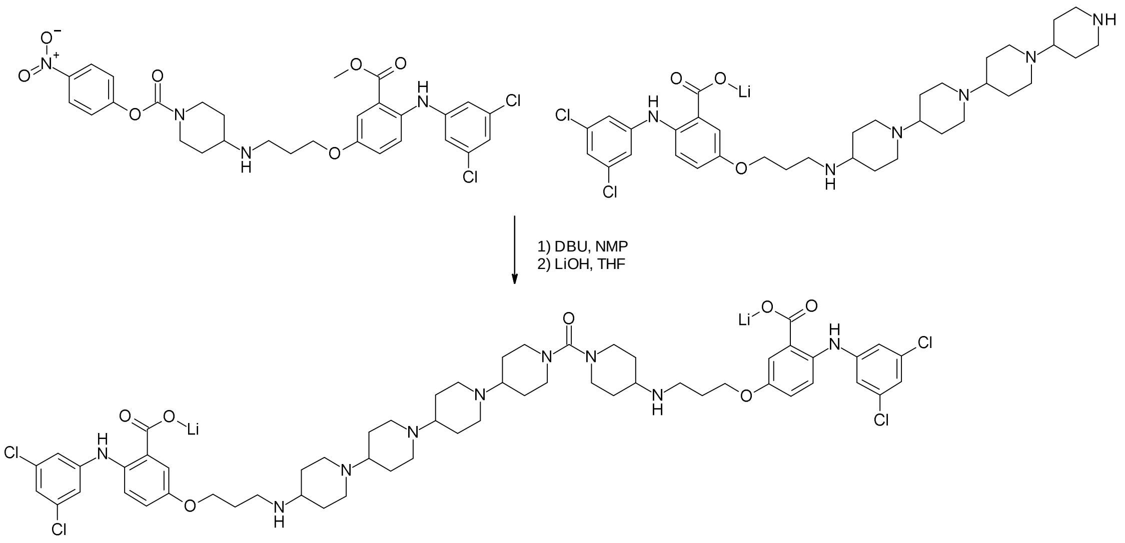

The compound was prepared in a similar manner to IIefrom 2-(3,5-dichlorophenylamino)-5-[3-([1,4';1',4";1",4"']quaterpiperidin-4-ylamino)-propoxy]benzoic acid methyl ester (70 mg, 0.1 mmol) and 4-{3-[4-(3,5-dichloro-phenylamino)-3methoxycarbonyl-phenoxy]-propylamino}-piperidine-1-carboxylic acid 4-nitro-phenyl ester (60 mg, 0.1 mmol) to give the title compound as colourless solid (19 mg). ESMS $[M+H]^+$ = 1150.65. ^{1}H NMR (300 MHz, MeOH-D4) δ: 7.55-7.60 (2H, m), 7.22-7.30 (2H, m), 6.90-7.11 (6H, m), 6.78 (2H, s), 4.02-4.15 (4H, m), 3.60-3.80 (4H, m),2.60-3.15 (13H, m), 2.35-2.59 (2H, m), 2.08-2.35 (8H, m), 1.75-2.05 (16H, m), 1.25-1.68 (10H, m).

Group I ligands: Amino acids and coupling agents were purchased from Novabiochem, and other reagents and solvents were purchased from Sigma-Aldrich or VWR. Succinimido-$(Pro)_5$-Gly-OH was synthesised by Fmoc peptide chemistry using a 3 fold molar excess of amino acid and HBTU, and 9 fold molar excess of DIPEA, on a Symphony automated peptide synthesiser (Protein Technologies). The peptide was cleaved using TFA:TIS:$H_2$0 95:2:2 and purified by RP-HPLC (C18 Gemini Axia, 5u 110A 250 x 21.2mm 20ml/min at 225 nm acetonitrile/water containing 0.1% TFA, Phemonenex).

Succinimido-$(Pro)_5$-Gly-OH was reacted to the methyl ester protected and amino functionalised 5-amino-2-(3,5-dichlorophenylamino)benzoic acid headgroup (Selcia Ltd) using a 2.5 molar excess of headgroup, BOP and DIPEA in DMF, and monitored by mass spectrometry for addition of headgroup to both ends of the peptide. The crude product was lyophilised and redisolved in methanol/water, containing the maximum water that still maintained a clear solution. The methyl ester was saponified by the addition of LiOH and the reaction monitored by mass spectrometry. The (Pro5) ligand was purified by RP-HPLC (C18 Gemini Axia as above) and analysed by LCMS (Agilent 1100). The series of polyproline ligands Pro6-9 were synthesised using the same method described above.
